# *Lactobacillus amylovorus* extracellular vesicles mitigate mammary gland ferroptosis via the gut-mammary gland axis

**DOI:** 10.1038/s41522-025-00752-4

**Published:** 2025-06-21

**Authors:** Qianzi Zhang, Dongpang Chen, Hanting Ding, Qihui Li, Siyu Yuan, Haobin Li, Wutai Guan, Shihai Zhang

**Affiliations:** 1 Guangdong Province Key Laboratory of Animal Nutrition Control, College of Animal Science, South China Agricultural University, Guangzhou, China; 2 College of Animal Science and National Engineering Research Center for Breeding Swine Industry, South China Agricultural University, Guangzhou, China; 3 Guangdong Laboratory for Lingnan Modern Agriculture, South China Agricultural University, Guangzhou, China

**Keywords:** Microbiota, Bacteria, Biofilms

## Abstract

Lactation is essential for supporting neonatal growth and development, and its regulation is influenced by the gut microbiota. However, the role of gut microbes in lactation under conditions of oxidative stress remains unclear. In this study, we identify a novel function for gut microbiota in regulating maternal lactation through the modulation of ferroptosis in the mammary gland under oxidative stress. We identify *Lactobacillus amylovorus* (*L. amylovorus*), enriched in mothers with low oxidative stress, as negatively correlating with both oxidative stress and ferroptosis. In a mouse model, *L. amylovorus* alleviates mammary ferroptosis and promotes lactation. In addition to producing of short-chain fatty acids, *L. amylovorus* secretes bacterial extracellular vesicles (BEVs) enriched in oleic acid, a monounsaturated fatty acid that can be transferred to the mammary gland. Mechanistically, the accumulation of oleic acid in mammary epithelial cells enhances their resistance to ferroptosis, thereby supporting milk production. These findings highlight the potential of *L. amylovorus* and its BEVs as therapeutic tools to counteract oxidative stress-induced lactation decline.

## Graphical abstract

**Figure:**
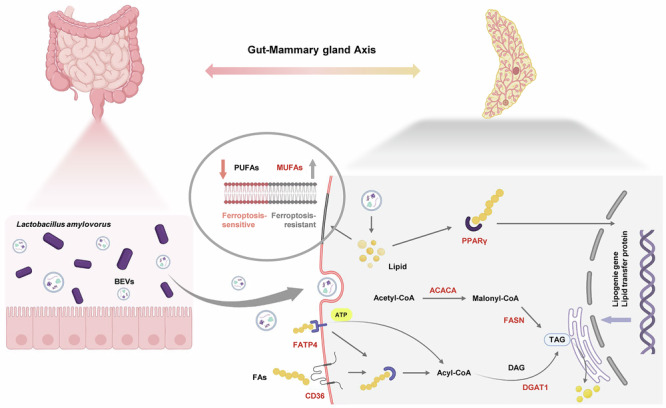
Bacterial extracellular vesicles released by *Lactobacillus amylovorus* in the maternal gut travel to the mammary gland via the gut-mammary gland axis. Upon reaching the mammary gland, they increase the proportion of monounsaturated fatty acids in mammary epithelial cell membranes, enhancing the resilience of these cells to ferroptosis. This reduction in ferroptosis improves milk lipid synthesis and transport, ultimately leading to higher milk fat production. The figure of proposed mechanisms is visualized using BioRender.

## Introduction

Oxidative stress plays a crucial role in maternal reproductive disorders and is strongly linked to negative pregnancy outcomes, including preeclampsia, gestational hypertension, gestational diabetes, preterm birth, and fetal growth restriction^1^. The lactation period, a critical phase in the maternal reproductive cycle, relies on the functional integrity of mammary epithelial cells to ensure normal milk production. Excessive oxidative stress can damage these cells, leading to reduced milk yield and altered milk composition, which negatively impacts the health of newborns.

Research has shown that host oxidative stress is closely linked to environmental factors^2^, dietary composition^3^, and different physiological stages, particularly pregnancy and lactation. In recent years, gut microbiota have also been identified as key players in the modulation of oxidative stress^4^. While substantial evidence indicates that gut microbiota primarily reside in the intestines and regulate local intestinal functions^5^, the relationship between gut microbiota, their metabolites, and mammary gland function remains largely unexplored. Some studies suggest that a small number of gut microbes can translocate to the mammary glands via the bloodstream, influencing mammary function and being transmitted to the next generation through milk^6^. However, such remote translocation is relatively rare due to the gut barrier. More commonly, gut microbiota affect distant organs by secreting metabolites that influence the function of the brain, liver, and heart^7–9^.

In addition to metabolites, bacterial extracellular vesicles (BEVs) serve as an important mechanism for long-distance communication by gut microbiota, garnering increasing research attention^10^. In mouse models, BEVs have been found to accumulate significantly in tissues such as joints and mammary glands, suggesting their regulatory roles in these organs. For instance, studies have demonstrated that *Fusobacterium nucleatum* in the gut can deliver BEVs to the joints, triggering local inflammation and thus contributing to exacerbating rheumatoid arthritis^11^.

In this study, we aimed to investigate whether the levels of *Lactobacillus amylovorus* (*L. amylovorus*) in the maternal gut are associated with oxidative stress and ferroptosis in the mammary glands. Further investigations revealed that *L. amylovorus* alleviates oxidative stress-induced ferroptosis by transferring oleic acid, a monounsaturated fatty acid, into mammary epithelial cells via BEVs. This enrichment enhances the synthesis capacity of milk lipids in the maternal mammary glands and improves the growth performance of offspring under oxidative stress conditions. These findings highlight the protective role of *L. amylovorus* in mitigating oxidative stress-induced ferroptosis and suggest its potential as a clinical therapeutic approach to improve maternal lactation decline caused by oxidative stress.

## Results

### Oxidative stress-driven ferroptosis impairs milk fat synthesis in lactating sows

**Fig. 1:**
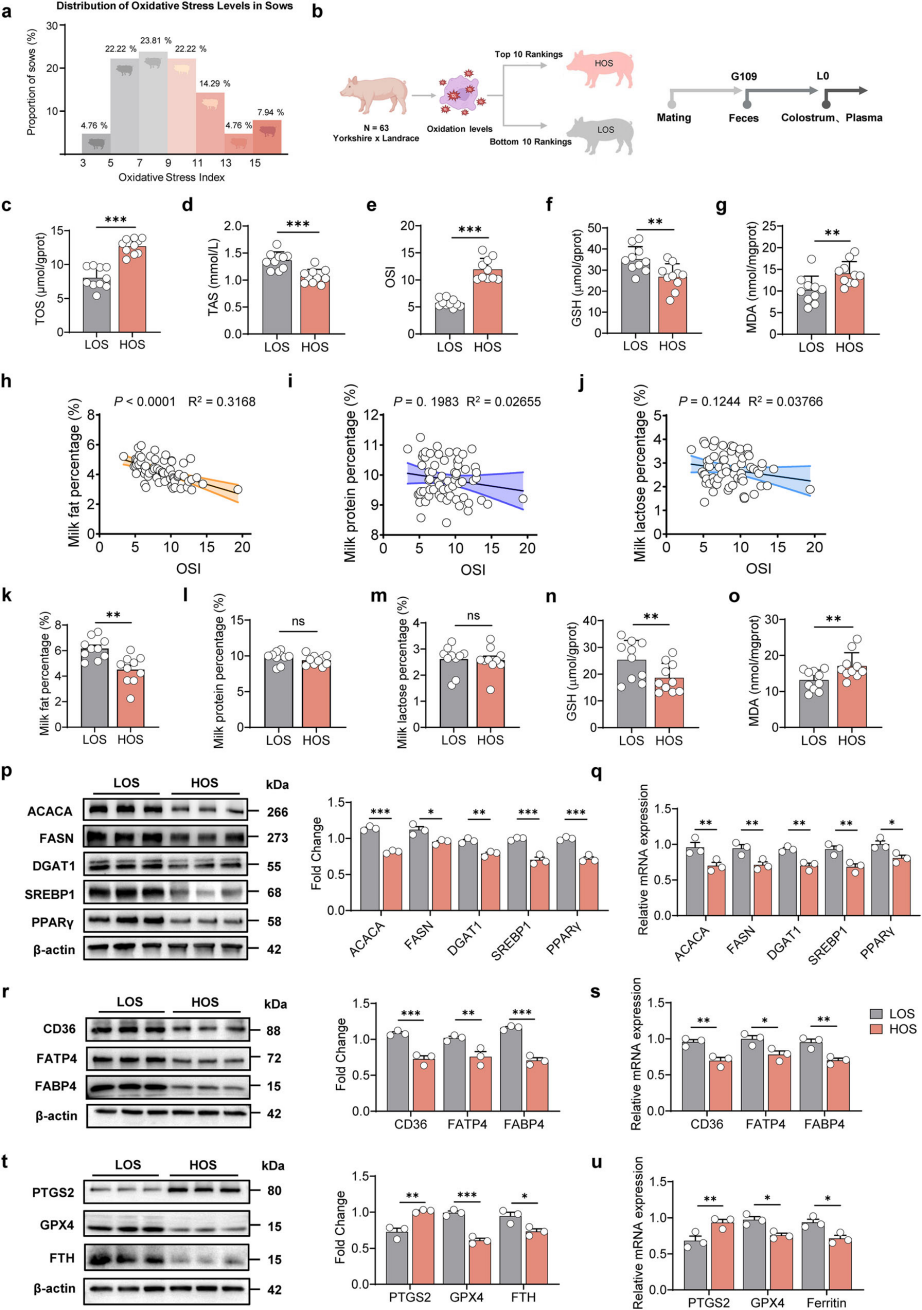
Oxidative stress induces ferroptosis in sow mammary glands and inhibits milk fat synthesis. **a** Distribution of oxidative stress levels in sows (*n* = 63). **b** Experimental grouping and sampling plan for sows, created with BioRender. **c** Total oxidative status in blood (*n* = 10). **d** Total antioxidant status in blood (*n* = 10). **e** Oxidative stress index in blood (*n* = 10). **f** Glutathione (GSH) levels in blood (*n* = 10). **g** Malondialdehyde (MDA) levels in blood (*n* = 10). Correlation between fat (**h**), protein (**i**), and lactose (**j**) contents and oxidative stress index (OSI) in colostrum (*n* = 63). Contents of fat (**k**), protein (**l**), and lactose (**m**) in colostrum (*n* = 10). Levels of GSH (**n**), MDA (**o**) in milk-derived somatic cells (*n* = 10). **p** Western blot analysis and fold change of milk fat synthesis-related proteins in colostrum-derived cells (*n* = 3). **q** mRNA expression of milk fat synthesis-related genes in colostrum-derived cells (*n* = 3). **r** Western blot analysis and fold change of milk fat transport-related proteins in colostrum-derived cells (*n* = 3). **s** mRNA expression of milk fat transport-related genes in colostrum-derived cells (*n* = 3). **t** Western blot analysis and fold change of ferroptosis-related proteins in colostrum-derived cells (*n* = 3). **u** mRNA expression of ferroptosis-related genes in colostrum-derived cells (*n* = 3). *P < 0.05; **P < 0.01; ***P < 0.001; ns not significant (*P* > 0.05). HOS sows under high oxidative stress, LOS sows under low oxidative stress.

Pigs serve as an excellent comparative model for studying human reproductive physiology because their mammary development, anatomical structure, and lactation mechanisms, particularly in milk fat synthesis and alveolar formation, closely resemble those in humans. In this study, we analyzed 63 recently farrowed sows based on their oxidative stress index (OSI) (Fig. 1a). The top and bottom 10 sows with the highest and lowest OSI were categorized into high oxidative stress (HOS) and low oxidative stress (LOS) groups, respectively, to assess the impact of oxidative stress on lactation (Fig. 1b). Significant differences were observed between the HOS and LOS groups in total oxidative stress (Fig. 1c), total antioxidant status (Fig. 1d), and the OSI (Fig. 1e) (*P* < 0.001). Blood analyses showed that sows in the HOS group had significantly lower levels of glutathione (GSH) (Fig. 1f) and malondialdehyde (MDA) (Fig. 1g) (*P* < 0.01).

Correlation analyses indicated a strong negative relationship between milk fat synthesis and OSI (*P* < 0.001), whereas milk protein and milk lactose levels were negatively correlated with OSI, but not significantly (*P* > 0.05) (Fig. 1h–j). The HOS group showed a significant reduction in milk fat content compared to the LOS group (*P* < 0.01), while milk protein and milk lactose levels did not differ significantly (*P* > 0.05) (Fig. 1k–m). To further investigate the effect of oxidative stress on milk fat synthesis, we isolated mammary cells from colostrum samples of sows in both groups. The HOS group exhibited significantly lower expression of key enzymes involved in lipid synthesis (ACACA, FASN, DGAT1), regulatory factors (SREBP1, PPARγ), and fatty acid transport proteins (CD36, FATP4, FABP4) compared to the LOS group (Fig. 1p, r) (*P* < 0.05). Similar reductions were observed at the gene expression level (Fig. 1q, s) (*P* < 0.05). These findings indicate that oxidative stress impairs lactational performance by decreasing the efficiency of milk fat synthesis and transport.

Oxidative stress is commonly associated with intracellular accumulation of ROS and disruption of iron homeostasis, both of which are key triggers of ferroptosis. Analysis of ferroptosis-related indicators revealed significant differences in GSH and MDA levels (Fig. 1n, o) (*P* < 0.01). The HOS group showed increased expression of ferroptosis marker genes and proteins, such as PTGS2, while the expression of genes and proteins involved in maintaining redox balance, including GPX4 and FTH, decreased (Fig. 1t, u) (*P* < 0.05). These results confirm that ferroptosis is significantly elevated in sows experiencing high oxidative stress. To further explore the connection between oxidative stress and ferroptosis, we developed an in vitro oxidative stress model using sow mammary epithelial cells. Treatment with ferroptosis inhibitor Fer-1 significantly reduced cell death caused by oxidative stress (Supplementary Fig. 1a–c) (*P* < 0.01). Additionally, intracellular reactive oxygen species (ROS) levels decreased markedly (Supplementary Fig. 1d, e), lipid peroxidation (LPO) was reversed (Supplementary Fig. 1f, g), and mitochondrial structure was preserved (Supplementary Fig. 1h) (*P* < 0.01). MDA concentrations decreased (Supplementary Fig. 1i), while GSH levels increased significantly (Supplementary Fig. 1j) (*P* < 0.01). The expression of ferroptosis marker genes (*PTGS2*) was downregulated, and genes (*Ferritin* and *GPX4*) involved in ferroptosis suppression were upregulated (Supplementary Fig. 1k) (*P* < 0.05). These results demonstrate that Fer-1 effectively alleviates ferroptosis induced by oxidative stress. Moreover, cells treated with Fer-1 showed larger intracellular lipid droplets (Supplementary Fig. 1l, m), higher extracellular and intracellular triglyceride levels (Supplementary Fig. 1n, o), and increased expression of genes involved in milk fat synthesis and transport (Supplementary Fig. 1p, q) (*P* < 0.05). These results indicate oxidative stress impairs mammary lipid synthesis and transport by increasing ferroptosis.

### Distinct gut microbiota profiles correlate with ferroptosis and milk fat synthesis in sows

**Fig. 2:**
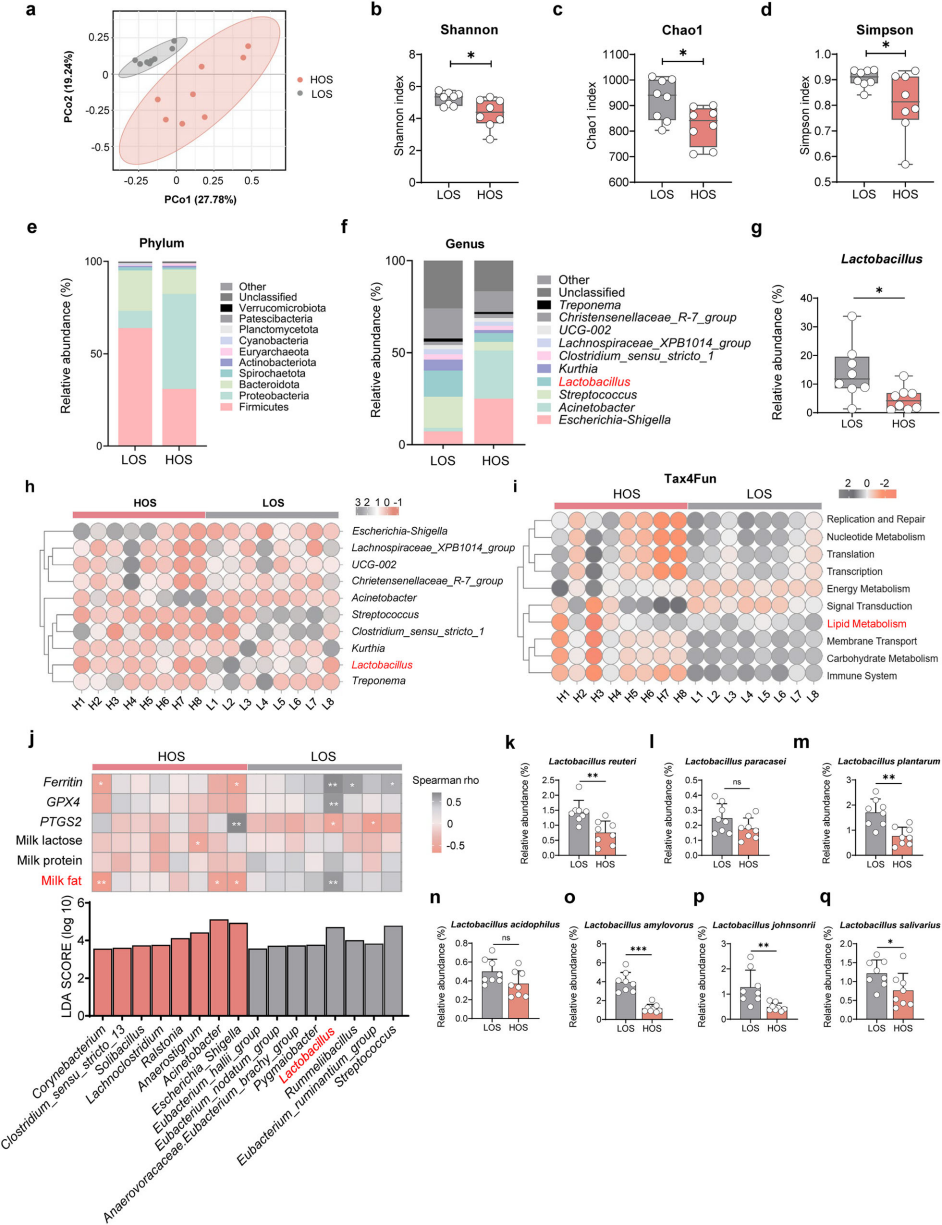
Maternal oxidative stress-induced ferroptosis and is closely associated with gut microbiota composition. **a** Principal Coordinate Analysis of gut microbiota composition (*n* = 8). Alpha diversity indices: Shannon (**b**), Chao1 (**c**), and Simpson (**d**) (*n* = 8). **e** Relative abundance of the top 10 bacterial phyla in fecal microbiota. **f** Relative abundance of the top 10 bacterial genera. **g** Comparison of *Lactobacillus* abundance between groups (*n* = 8). **h** Distribution of microbial genera across individual sows (*n* = 8). **i** Functional prediction of microbiota using Tax4Fun analysis (*n* = 8). **j** Linear Discriminant Analysis identifying differentially abundant bacteria between HOS and LOS groups (LDA > 3.5, *P* < 0.05) and Spearman’s correlations between selected bacteria, milk components, and ferroptosis genes. **k**–**q** Relative abundance of dominant *Lactobacillus* species in the intestinal microbiota of sows (*n* = 8). *P < 0.05; **P < 0.01; ***P < 0.001; ns, not significant (*P* > 0.05). HOS sows under high oxidative stress, LOS Sows under low oxidative stress.

To examine whether gut microbiota affect oxidative stress, ferroptosis, and milk fat synthesis and transport, we compared the fecal microbiota of sows in the HOS group with those in the LOS group. Principal Coordinate Analysis (PCoA) revealed distinct β-diversity patterns between the two groups (Fig. 2a), and notable differences in α-diversity (Shannon, Chao1, Simpson Indices) were also observed (Fig. 2b–d) (*P* < 0.05). At the phylum level, the Firmicutes-to-Bacteroidetes ratio was significantly higher in the LOS group, suggesting that these sows were generally healthier than those in the HOS group (Fig. 2e).

Further genus-level analysis showed a markedly higher abundance of *Lactobacillus* in the LOS group (Fig. 2f, g) (*P* < 0.05), a finding corroborated by a differential abundance heatmap (Fig. 2h). Functional profiling using Tax4Fun indicated that the LOS group exhibited stronger associations with lipid metabolism (Fig. 2i). Moreover, Spearman correlation analysis of the relative abundances of different genera against key indicators in colostrum, such as milk fat, lactose, protein content, and ferroptosis-related genes expression, demonstrated that *Lactobacillus* was positively correlated with enhanced milk fat synthesis and lower ferroptosis levels (Fig. 2j) (*P* < 0.05).

To identify the predominant *Lactobacillus* species in these sows, we conducted quantitative assessments of the seven most abundant *Lactobacillus* species in the pig gut^12^. Among them, *Lactobacillus reuteri*, *Lactobacillus plantarum*, *Lactobacillus amylovorus*, *Lactobacillus johnsonii*, and *Lactobacillus salivarius* were significantly enriched in the LOS group (Fig. 2k–q) (*P* < 0.05), suggesting that these species hold strong potential for reducing oxidative stress and ferroptosis while promoting milk fat synthesis.

### Gut microbiota transplantation from sows modulates ferroptosis and milk fat synthesis in mice

**Fig. 3:**
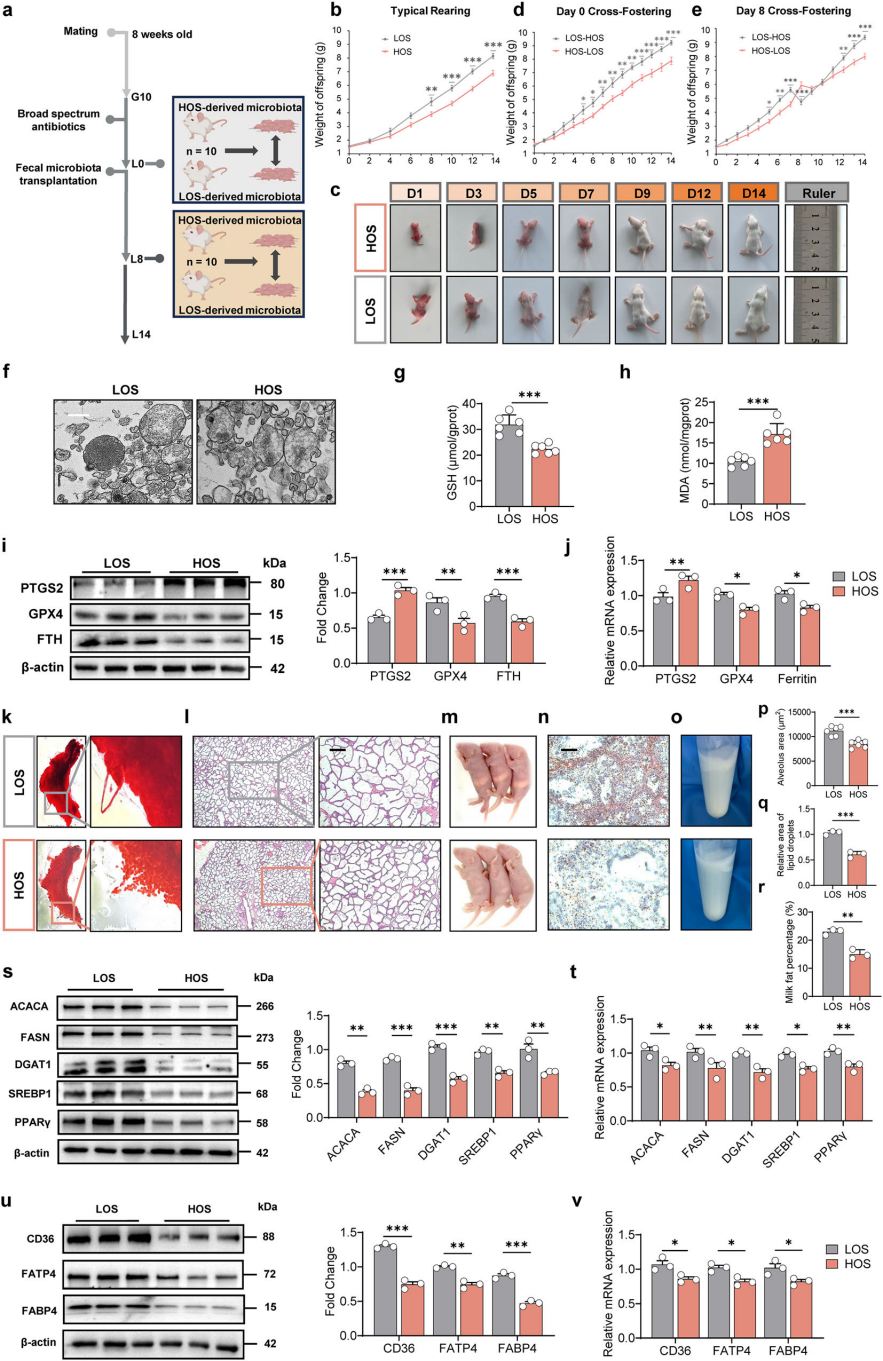
Impact of maternal gut microbiota transplantation on ferroptosis in mammary glands and lactation performance. **a** Experimental design for fecal microbiota transplantation in mice, created with BioRender. **b** Body weights of pups under typical rearing (*n* = 6). Litter sizes were adjusted to 13 for each mother on L0. **c** Offspring development metrics. Body weights of pups under different rearing conditions: **d** Day 0 cross-fostering, **e** Day 8 cross-fostering group (*n* = 6). Litter sizes were adjusted to 13 for each mother on L0. **f** Mitochondrial morphology analysis. Scale bar: 500 nm. Levels of glutathione (GSH) (**g**), malondialdehyde (MDA) (**h**) in mouse blood (*n* = 6). **i** Western blot analysis and fold change of ferroptosis-related proteins in mouse mammary gland tissue (*n* = 3). **j** mRNA expression of ferroptosis-related genes in mammary gland tissue of mice (*n* = 3). **k** Carmine-stained whole-mount analysis of mammary glands on lactation day 14. **l** Histological sections of mammary glands stained with hematoxylin and eosin on lactation day 14. Scale bar: 200 μm. **m** Milk clot size in the stomachs of mouse pups (*n* = 3). **n** Oil Red O staining of lipids in mammary gland sections. Scale bar: 200 μm. **o** Milk fat layer after centrifugation. Quantitative analysis of alveolar area (**p**), lipid droplet area (**q**), and milk fat percentage (**r**) (*n* = 3). **s** Western blot analysis and fold change of milk fat synthesis-related proteins in mouse mammary gland tissue (*n* = 3). **t** mRNA expression of milk fat synthesis-related genes in mammary gland tissue of mice (*n* = 3). **u** Western blot analysis and fold change of milk fat transport-related proteins in mouse mammary gland tissue (*n* = 3). **v** mRNA expression of milk fat transport-related genes in mammary gland tissue of mice (*n* = 3). *P < 0.05; **P < 0.01; ***P < 0.001; ns not significant (*P* > 0.05). HOS mice under high oxidative stress, LOS mice under low oxidative stress.

To determine whether gut microbiota regulate milk fat synthesis by influencing oxidative stress and ferroptosis, we conducted an in vivo experiment by transplanting fecal microbiota from sows in the HOS and LOS groups into mice, respectively (Fig. 3a). On day 14 post-transplantation, we analyzed the cecal microbiota of the dams. The LOS group formed a distinct cluster in β-diversity analysis (Supplementary Fig. 2a) and differed in α-diversity (Supplementary Fig. 2b–d). At the phylum level, the LOS group showed a higher Firmicutes-to-Bacteroidetes ratio (Supplementary Fig. 2e). At the genus level, *Lactobacillus* abundance was notably higher in the LOS group (Supplementary Fig. 2f, g) (*P* < 0.001), mirroring the composition observed in the transplanted sow microbiota, with *Lactobacillus amylovorus* as the most prevalent species. Similarly, among seven common *Lactobacillus* species, *Lactobacillus amylovorus*, *Lactobacillus plantarum*, *Lactobacillus johnsonii*, and *Lactobacillus reuteri* were significantly more abundant in the LOS group (Supplementary Fig. 2j–p) (*P* < 0.01). And the Spearman analysis revealed that, similar to the results from the sow experiment, the *Lactobacillus* had a strong association with the iron-related death genes, such as *PTGS2*, *GPX4*, and *Ferritin* (Supplementary Fig. 2h) (*P* < 0.05). These results indicate that the microbiota transplantation from sows to mice was successful.

The experiment lasted from day 0 of lactation to day 14, the peak of lactation, in the mice. We standardized the litter size to 13 pups per dam at birth and adjusted pup weights to ensure uniform growth. Under these conditions, pups in the HOS group had significantly lower body weights than those in the LOS group (Fig. 3b), and differences in body size between the two groups were readily visible (Fig. 3c). To minimize individual and environmental variability, we conducted cross-fostering starting on lactation day 0 and day 8. Pups cross-fostered on day 0 showed similar growth trends to those in the non-cross-fostered group, indicating minimal impact from external factors (Fig. 3d). This suggests that differences in offspring growth were mainly shaped by the maternal microbiota introduced by HOS or LOS sows. Moreover, pups cross-fostered on day 8 revealed that dams carrying LOS-derived microbiota produced more milk, supporting faster growth in their offspring (Fig. 3e).

We next examined how microbial transfer affected ferroptosis in the dams by analyzing their mammary gland tissues. Transmission electron microscopy showed extensive mitochondrial damage in the HOS group (Fig. 3f). These dams also had reduced GSH content (Fig. 3g) and elevated MDA levels (Fig. 3h) (*P* < 0.001), all indicative of enhanced ferroptosis. Ferroptosis marker genes and proteins (e.g., *PTGS2*) were upregulated, while genes (*GPX4* and *Ferritin*) involved in maintaining redox balance were downregulated (Fig. 3i, j) in the mammary gland (*P* < 0.05), confirming that HOS-derived microbiota significantly increased ferroptosis in the dams.

To compare mammary gland development in the HOS and LOS groups, we performed whole-mount and H&E staining. LOS dams displayed more densely packed secretory lobules (Fig. 3k) and larger alveolus areas (Fig. 3l, p) (*P* < 0.001), both favorable for milk secretion and storage. Pups from LOS dams also showed larger gastric milk curds (Fig. 3m), suggesting greater milk intake and production. Oil Red O staining (Fig. 3n, q) and measurement of the milk fat percentage (Fig. 3o, r) further confirmed that LOS dams produced milk with higher lipid content (*P* < 0.01), promoting faster pup growth. At the molecular level, key enzymes and regulatory factors for lipid synthesis (ACACA, FASN, DGAT1, SREBP1, PPARγ) as well as fatty acid transporters (CD36, FATP4, FABP4) were significantly upregulated in the mammary gland of LOS group (Fig. 3s–v) (*P* < 0.05). Intriguingly, Tax4Fun analysis linked the LOS microbiota to stronger lipid metabolism functions (Supplementary Fig. 2i). These findings indicate that compared with HOS-derived microbiota, LOS-derived microbiota protected the dams from ferroptosis and promoted milk fat synthesis and transport in the mammary gland.

### LOS-derived microbiota alleviates ferroptosis and maintains mammary lactation under oxidative stress

**Fig. 4:**
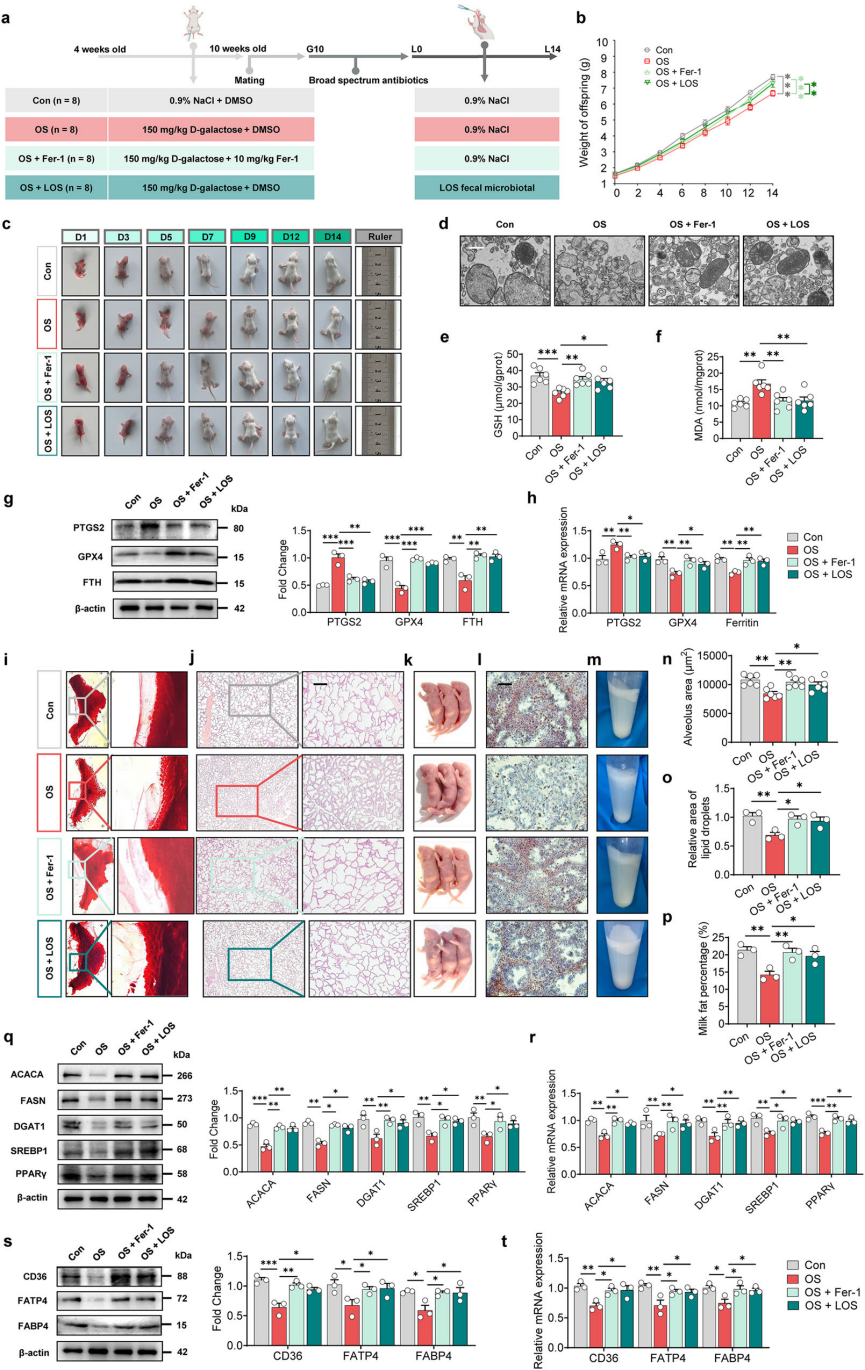
Maternal microbiota transplantation reduces ferroptosis in mammary glands and enhances lactation performance under oxidative stress. **a** Experimental design for fecal microbiota transplantation to mitigate oxidative stress in mice, created with BioRender. **b** Body weights of pups under typical rearing (*n* = 6). Litter sizes were adjusted to 13 for each mother on L0. **c** Offspring development metrics. **d** Mitochondrial morphology analysis. Scale bar: 500 nm. Levels of glutathione (GSH) (**e**), malondialdehyde (MDA) (**f**) in mouse blood (*n* = 6). **g** Western blot analysis and fold change of ferroptosis-related proteins in mouse mammary gland tissue (*n* = 3). **h** mRNA expression of ferroptosis-related genes in mammary gland tissue of mice (*n* = 3). **i** Carmine-stained whole-mount analysis of mammary glands on lactation day 14. **j** Histological sections of mammary glands stained with hematoxylin and eosin on lactation day 14. Scale bar: 200 μm. **k** Size of milk clots in the stomachs of mouse pups (*n* = 3). **l** Oil Red O staining of milk fat in mammary gland sections. Scale bar: 200 μm. **m** Milk fat layer after centrifugation. Quantitative analysis of alveolar area (**n**), lipid droplet area (**o**), and milk fat percentage (**p**) (*n* = 3). **q** Western blot analysis and fold change of milk fat synthesis-related proteins in mouse mammary gland tissue (*n* = 3). **r** mRNA expression of milk fat synthesis-related genes in mammary gland tissue of mice (*n* = 3). **s** Western blot analysis and fold change of milk fat transport-related proteins in mouse mammary gland tissue (*n* = 3). **t** mRNA expression of milk fat transport-related genes in mammary gland tissue of mice (*n* = 3). *P < 0.05; **P < 0.01; ***P < 0.001; ns not significant (*P* > 0.05). LOS sows under low oxidative stress.

To determine whether LOS-derived microbiota protect mammary glands from ferroptosis and impaired lactation under oxidative stress, we established an oxidative stress model in mice. We then treated the mice either with a ferroptosis inhibitor or with fecal microbiota transplanted from LOS sows (Fig. 4a). To accurately assess lactation, we standardized each litter size to 13 pups and ensured uniform pup weights at birth. Both ferroptosis inhibition and LOS microbiota transplantation markedly reduced oxidative stress, evidenced by steady weight gain (Fig. 4b) and clear physical improvements compared with the oxidative stress (OS) group (Fig. 4c). Consistent with these outcomes, mice treated with ferroptosis inhibitors or LOS microbiota had higher GSH levels (Fig. 4e) and lower MDA levels (Fig. 4f) (*P* < 0.05). Furthermore, mitochondrial structure was better preserved (Fig. 4d). The expression of ferroptosis marker PTGS2 (both protein and gene) declined, while redox-regulating genes and proteins (GPX4 and FTH) were upregulated (Fig. 4g, h) (*P* < 0.05), indicating a substantial reduction in ferroptosis.

Mice in the ferroptosis inhibitor and LOS microbiota groups also had healthier mammary glands with a higher abundance of milk fat. These improvements were reflected by more compact alveolar structures (Fig. 4i), larger alveolus areas (Fig. 4j, n) (*P* < 0.05), and greater milk production (Fig. 4k). Oil Red O staining (Fig. 4l, o) and milk fat percentage measurements (Fig. 4m, p) confirmed higher fat content in these groups (*P* < 0.05). Additionally, enzymes involved in lipid synthesis (ACACA, FASN, DGAT1), key transcription factors (SREBP1, PPARγ), and fatty acid transporters (CD36, FATP4, FABP4) were significantly upregulated in the ferroptosis inhibitor and LOS microbiota groups (Fig. 4q–t) (*P* < 0.05). Taken together, these findings demonstrate that supplementing mice with LOS-derived microbiota alleviates ferroptosis and preserves milk fat synthesis in the mammary gland under oxidative stress.

### *L. amylovorus* emerges as the key LOS-derived *Lactobacillus* for counteracting oxidative stress-induced ferroptosis and supporting milk fat synthesis

To identify which *Lactobacillus* species in the LOS group efficiently alleviates oxidative stress-induced ferroptosis and improves milk fat synthesis, we established an in vitro oxidative stress model in HC11 mammary epithelial cells using H_2_O_2_. We then prepared cell-free supernatants from five *Lactobacillus* species (*Lactobacillus amylovorus*, *Lactobacillus johnsonii*, *Lactobacillus reuteri*, *Lactobacillus plantarum*, and *Lactobacillus salivarius*) enriched in the LOS group and screened their antioxidant and anti-ferroptosis effects (Supplementary Fig. 3a). Among these five species, *L. amylovorus* demonstrated the strongest ability to reduce excessive free radicals and lipid peroxides induced by oxidative stress (Supplementary Fig. 3b–e) (*P* < 0.01). Furthermore, *L. amylovorus* supernatant significantly increased the lipid droplet content secreted by mammary epithelial cells compared with the other four species, as evidenced by larger lipid droplet areas after Oil Red O staining (Supplementary Fig. 3f, g) and higher extracellular and intracellular triglyceride levels (Supplementary Fig. 3h, i) (*P* < 0.05). These findings suggest that *L. amylovorus* may be a key gut microbe for mitigating ferroptosis induced by oxidative stress and enhancing lactation capacity.

### *L. amylovorus* extracellular vesicles as primary mediators of protection against oxidative stress-induced ferroptosis

**Fig. 5:**
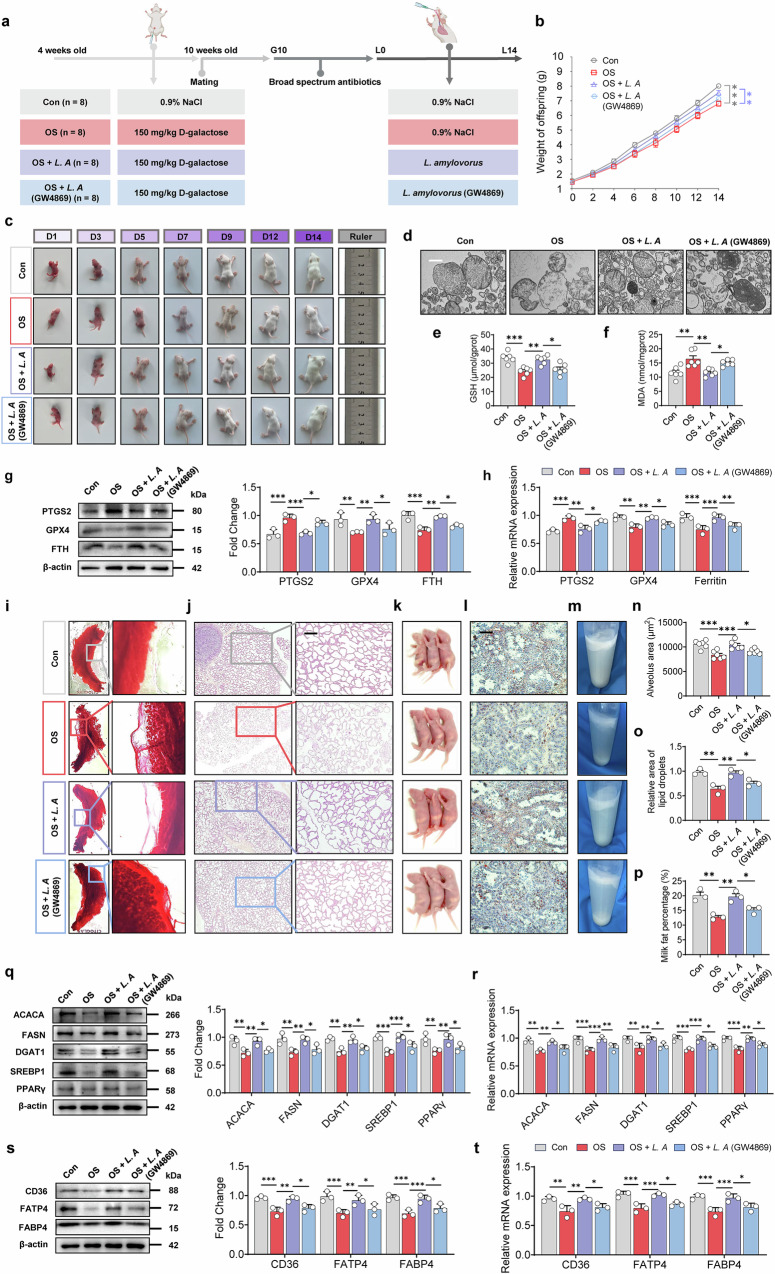
*Lactobacillus amylovorus* reduces oxidative stress-induced ferroptosis in mammary glands and enhances lactation performance via metabolites and BEVs. **a** Experimental design for *Lactobacillus amylovorus* transplantation to alleviate oxidative stress in mice, created with BioRender. **b** Body weights of pups (*n* = 6). Litter sizes were adjusted to 13 for each mother on L0. **c** Offspring development metrics. **d** Mitochondrial morphology analysis. Scale bar: 500 nm. Levels of glutathione (GSH) (**e**), malondialdehyde (MDA) (**f**) in mouse blood (*n* = 6). **g** Western blot analysis and fold change of ferroptosis-related proteins in mouse mammary gland tissue (*n* = 3). **h** mRNA expression of ferroptosis-related genes in mammary gland tissue of mice (*n* = 3). **i** Carmine-stained whole-mount analysis of mammary glands on lactation day 14. **j** Histological sections of mammary glands stained with hematoxylin and eosin on lactation day 14. Scale bar: 200 μm. **k** Size of milk clots in the stomachs of mouse pups (*n* = 3). **l** Oil Red O staining of milk fat in mammary gland sections. Scale bar: 200 μm. **m** Milk fat layer after centrifugation. Quantitative analysis of alveolar area (**n**), lipid droplet area (**o**), and milk fat percentage (**p**) (*n* = 3). **q** Western blot analysis and fold change of milk fat synthesis-related proteins in mouse mammary gland tissue (*n* = 3). **r** mRNA expression of milk fat synthesis-related genes in mammary gland tissue of mice (*n* = 3). **s** Western blot analysis and fold change of milk fat transport-related proteins in mouse mammary gland tissue (*n* = 3). **t** mRNA expression of milk fat transport-related genes in mammary gland tissue of mice (*n* = 3). *P < 0.05; **P < 0.01; ***P < 0.001; ns not significant (*P* > 0.05). *L. A* *Lactobacillus amylovorus* cell-free supernatant, *L. A* (GW4869) *Lactobacillus amylovorus* cell-free supernatant with GW4869-mediated bacterial extracellular vesicle depletion.

To determine whether *Lactobacillus amylovorus* protects mammary glands from oxidative stress, we conducted an in vivo mouse experiment. Previous research has shown that gut microbes can influence distant tissues and organs by releasing bioactive factors, including bacterial extracellular vesicles. We hypothesized that *L. amylovorus* exerts its effects primarily through metabolites and/or vesicle secretion. In this study, the extracellular vesicle inhibitor GW4869 was used to assess whether inhibiting *L. amylovorus* vesicle release diminishes its protective effects on ferroptosis and milk fat synthesis (Fig. 5a and Supplementary Fig. 4). The results from in vivo treatment of mice with the GW4869 inhibitor alone show that GW4869 has no significant effect on offspring growth performance (Supplementary Fig. 5a–c), maternal mammary ferroptosis levels (Supplementary Fig. 5d–g), or lactation performance (Supplementary Fig. 5h–q) (*P* > 0.05). Similarly, in vitro treatment of HC11 cells with GW4869 inhibitor also reveals no significant impact on ferroptosis (Supplementary Fig. 6a–d) or milk fat synthesis (Supplementary Fig. 6e–h) in mammary epithelial cells (*P* > 0.05). These findings collectively indicate that host-derived BEVs do not contribute to the observed phenotypes.

Quantification of *L. amylovorus* in the maternal gut confirmed successful colonization of the administered strain (Supplementary Fig. 7). In vivo experiments further revealed that *L. amylovorus* significantly mitigated oxidative stress-induced damage in pups, and this benefit was greatly reduced upon vesicle inhibition (Fig. 5b, c). As illustrated in Fig. 5d, mice gavaged with *L. amylovorus* exhibited more intact mitochondrial structures in their mammary glands, whereas mice treated with *L. amylovorus* with GW4869 continued to show severe mitochondrial damage. This trend was consistent with changes in GSH and MDA in the blood (Fig. 5e, f) (*P* < 0.05). Ferroptosis-related assessments (Fig. 5g, h) indicated that mice receiving *L. amylovorus* had substantially lower ferroptosis compared with the oxidative stress group (*P* < 0.05). While partial protection remained in the vesicle-inhibited group, it was noticeably weaker than the normal *L. amylovorus* treatment.

Similarly, mice supplemented with *L. amylovorus* had tightly arranged alveoli (Fig. 5i), larger alveolus areas (Fig. 5j, n), increased pup milk intake (Fig. 5k), and more lipid droplet secretion (Fig. 5l, o) (*P* < 0.05). Centrifugation of milk samples revealed a higher milk fat percentage in the *L. amylovorus* group (Fig. 5m, p) (*P* < 0.05). Enzymes and genes involved in milk fat synthesis and transport were significantly upregulated (Fig. 5q–t) (*P* < 0.05), demonstrating *L. amylovorus*’s capacity to alleviate oxidative stress-induced damage to milk fat synthesis. While minor improvement persisted with bacterial extracellular vesicle inhibition, it was markedly reduced compared with complete *L. amylovorus* treatment. These results, consistent with previous in vitro findings, suggest that *L. amylovorus* combats oxidative stress-induced ferroptosis and maintains milk fat synthesis largely through bacterial extracellular vesicle secretion, while additional metabolites may also play a role.

In order to further validate the function of *L. amylovorus* BEVs, we isolated and purified them by ultracentrifugation (Supplementary Fig. 8a). Transmission electron microscopy revealed that the vesicles had a disc-like shape with an average diameter of about 100 nm (Supplementary Fig. 8b), and nanoparticle tracking analysis (NTA) confirmed a predominant size of approximately 111.6 nm (Supplementary Fig. 8c). After labeling these vesicles with Dil dye, we co-cultured them with HC11 mouse mammary epithelial cells. Fluorescence microscopy showed that they entered the cells and accumulated around the nucleus (Supplementary Fig. 8d). In an H_2_O_2_-induced oxidative stress model in HC11 cells, *L. amylovorus* BEVs significantly reduced ROS (Supplementary Fig. 9a, b) and LPO levels (Supplementary Fig. 9c, d) (*P* < 0.05), providing protection comparable to that of ferroptosis inhibitors and *L. amylovorus* cell-free supernatant. *L. amylovorus* BEVs also increased lipid droplet formation (Supplementary Fig. 9e, f) and both extracellular and intracellular triglyceride levels (Supplementary Fig. 9g, h) (*P* < 0.05). In contrast, *L. amylovorus* pretreated with GW4869 (a vesicle secretion inhibitor) showed a markedly weaker protective effect, consistent with our in vivo findings. Short-chain fatty acids (SCFAs) are another major metabolite of *L. amylovorus* and have been reported to mitigate ferroptosis. We hypothesized that SCFAs may partly alleviate oxidative stress in mammary epithelial cells when BEV secretion is inhibited. Treatment with SCFA mixture (acetic, propionic, and butyric acids in a 65:20:15 ratio) decreased ROS (Supplementary Fig. 10a, b) and LPO (Supplementary Fig. 10c, d), while enhancing triglyceride levels (Supplementary Fig. 10e, f) and lipid droplet formation (Supplementary Fig. 10g, h). However, no statistically significant differences were observed (*P* > 0.05). Although SCFAs exhibited some protective effects, they provided less protection compared to *L. amylovorus* BEVs. Collectively, these in vitro results support the conclusion that *L. amylovorus* mitigates oxidative stress-induced ferroptosis primarily through BEV secretion, with a smaller contribution from SCFAs alone.

### *L. amylovorus* BEVs mitigate oxidative stress-induced ferroptosis and preserve mammary function in mice

**Fig. 6:**
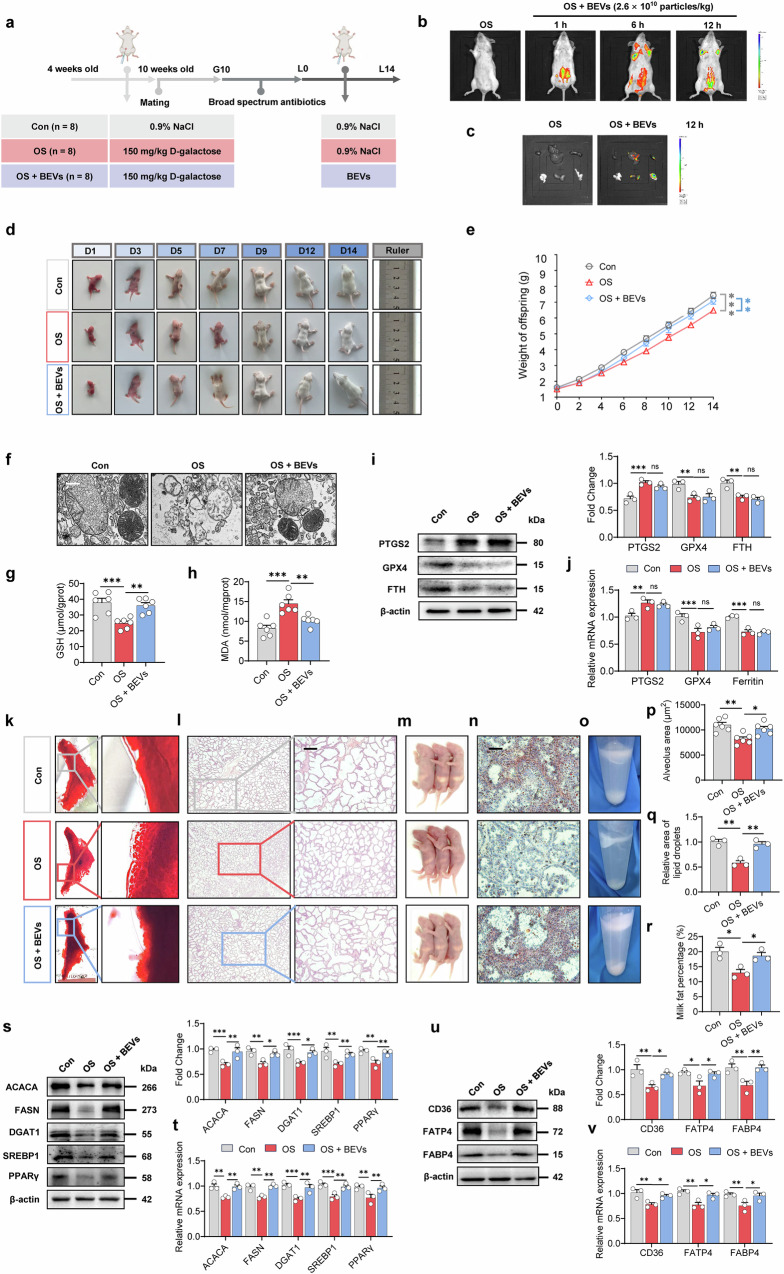
*Lactobacillus amylovorus* alleviates oxidative stress-induced ferroptosis in mouse mammary glands and enhances lactation performance via bacterial extracellular vesicles. **a** Experimental design for evaluating the protective effect of *Lactobacillus amylovorus* extracellular vesicles against oxidative stress in mice, created with BioRender. In vivo imaging (**b**) showed BEV localization in the body, and ex vivo imaging (**c**) revealed their distribution in the mammary gland. **d** Assessment of offspring development. **e** Body weights of pups (*n* = 6). Litter sizes were adjusted to 13 for each mother on L0. **f** Mitochondrial morphology analysis. Scale bar: 500 nm. Levels of glutathione (GSH) (**g**), malondialdehyde (MDA) (**h**) in mouse blood (*n* = 6). **i** Western blot analysis and fold change of ferroptosis-related proteins in mouse mammary gland tissue (*n* = 3). **j** mRNA expression of ferroptosis-related genes in mammary gland tissue of mice (*n* = 3). **k** Carmine-stained whole-mount analysis of mammary glands on lactation day 14. **l** Histological sections of mammary glands stained with hematoxylin and eosin on lactation day 14. Scale bar: 200 μm. **m** Size of milk clots in the stomachs of mouse pups (*n* = 3). **n** Oil Red O staining of milk fat in mammary gland sections. Scale bar: 200 μm. **o** Milk fat layer after centrifugation. Quantitative analysis of alveolar area (**p**), lipid droplet area (**q**), and milk fat percentage (**r**) (*n* = 3). **s** Western blot analysis and fold change of milk fat synthesis-related proteins in mouse mammary gland tissue (*n* = 3). **t** mRNA expression of milk fat synthesis-related genes in mammary gland tissue of mice (*n* = 3). **u** Western blot analysis and fold change of milk fat transport-related proteins in mouse mammary gland tissue (*n* = 3). **v** mRNA expression of milk fat transport-related genes in mammary gland tissue of mice (*n* = 3). *P < 0.05; **P < 0.01; ***P < 0.001; ns not significant (*P* > 0.05).

To evaluate the effectiveness of *L. amylovorus* BEVs in vivo, we directly treated BEVs into mice under oxidative stress to assess their impact on ferroptosis and mammary gland function (Fig. 6a). We first confirmed whether these vesicles could reach the mammary gland by labeling them with Dil dye and administering them via intraperitoneal injection. In vivo imaging at 1, 6, and 12 h revealed that fluorescence initially remained near the injection site but later appeared in both the abdominal cavity and mammary glands (Fig. 6b). Dissection and organ imaging at 12 h further confirmed fluorescence signals in the mammary gland (Fig. 6c), indicating that *L. amylovorus* BEVs can circulate to the mammary gland.

Under oxidative stress, mice receiving *L. amylovorus* BEVs showed marked improvements in mammary function, as evidenced by higher pup weights (Fig. 6e) and larger overall size (Fig. 6d). These mice also displayed intact mitochondrial structures in the mammary gland (Fig. 6f), increased levels of GSH (Fig. 6g), and reduced levels of MDA (Fig. 6h) (*P* < 0.01). Surprisingly, ferroptosis-related proteins (Fig. 6i) and genes (Fig. 6j) did not change significantly (*P* > 0.05), suggesting that BEVs reduce ferroptosis through mechanisms beyond direct gene or protein regulation. In addition, BEV-treated mice exhibited tighter alveolar structures (Fig. 6k), larger alveolus areas (Fig. 6l, p), increased milk content in pups (Fig. 6m), elevated lipid droplet formation (Fig. 6n, q), and higher milk fat percentage (Fig. 6o, r) (*P* < 0.05). Genes and proteins linked to milk fat synthesis and transport were also significantly upregulated (Fig. 6s–v) (*P* < 0.05). These findings illustrate that *L. amylovorus* BEVs substantially mitigate oxidative stress-induced ferroptosis, thereby preserving milk fat synthesis and transport.

### Lipid components of *L. amylovorus* BEVs govern ferroptosis protection and milk fat preservation

**Fig. 7:**
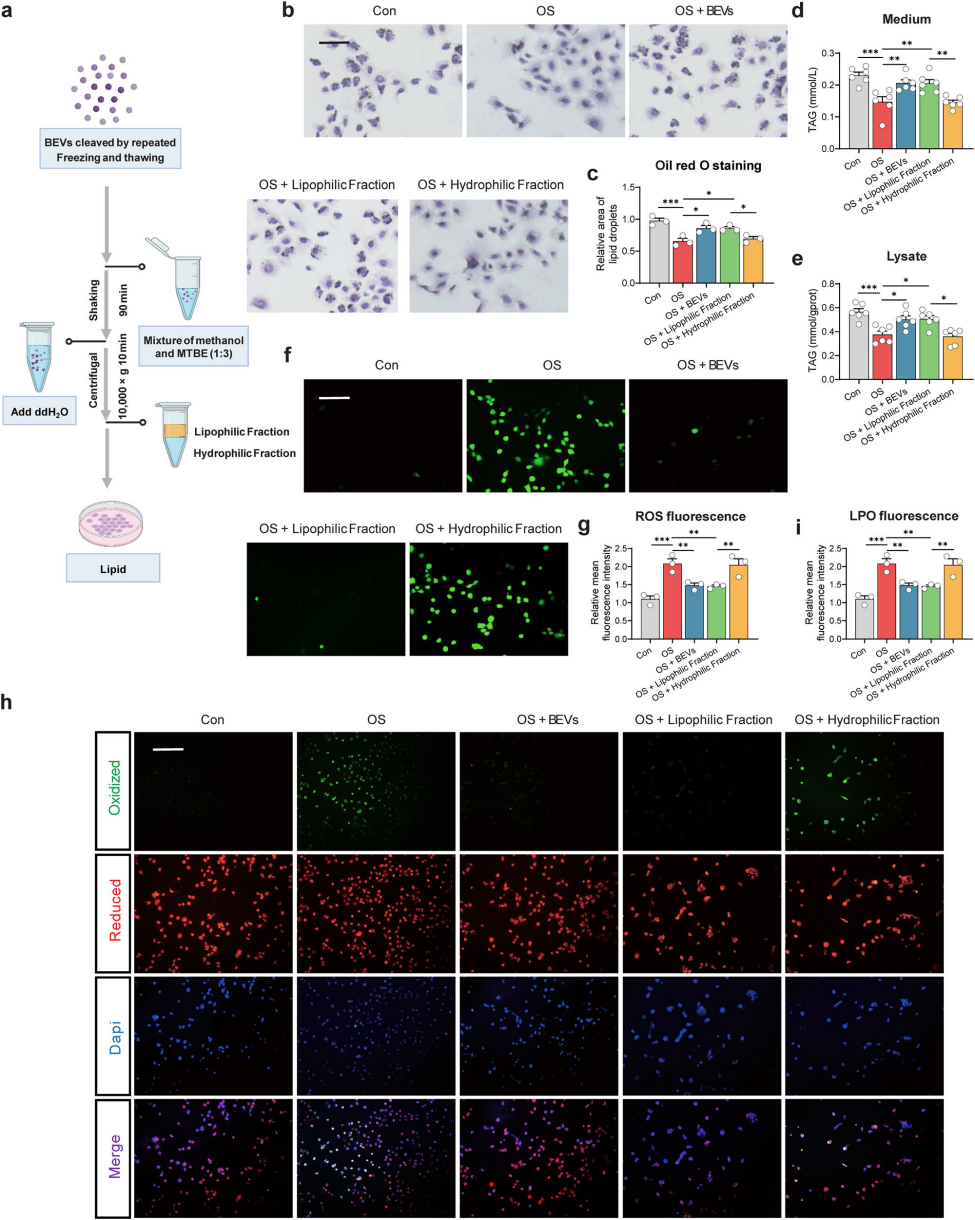
*Lactobacillus amylovorus* BEVs mitigate oxidative stress-induced ferroptosis and enhance milk fat synthesis through lipophilic substances. **a** Experimental design for extracting lipids from BEVs, created with BioRender. **b** Oil Red O staining of HC11. Scale bar: 100 μm. **c** Quantitative analysis of Oil Red O staining (*n* = 3). Triglyceride concentrations in the medium (**d**) and within cells (**e**) (*n* = 6). **f** Detection of intracellular reactive oxygen species (ROS) levels using ROS fluorescence assay. Scale bar: 200 μm. **g** Relative mean fluorescence intensity of ROS (*n* = 3). **h** Detection of intracellular lipid peroxidation (LPO) levels using LPO fluorescence assay. Scale bar: 500 μm. **i** Relative mean fluorescence intensity of LPO (*n* = 3). *P < 0.05; **P < 0.01; ***P < 0.001; ns not significant (*P* > 0.05).

Having confirmed that *L. amylovorus* BEVs alleviate oxidative stress-induced ferroptosis and enhance mammary function, we next examined their underlying mechanism. Since BEVs are composed of proteins, lipids, and nucleic acids, we investigated which biomolecules drive their protective effects. First, we treated the BEVs with DNase I, RNase A, or Proteinase K, then examined their ability to mitigate ferroptosis in oxidative stress-induced HC11 cells (Supplementary Fig. 11a). Removing nucleic acids or proteins did not diminish *L. amylovorus* BEV-mediated protection, as indicated by unchanged ROS (Supplementary Fig. 11b, c), LPO (Supplementary Fig. 11d, e), lipid droplet formation (Supplementary Fig. 11f, g), and extracellular/intracellular triglyceride levels (Supplementary Fig. 11h, i) (*P* > 0.05). These results suggest that nucleic acids and proteins do not play the primary protective role, prompting us to focus on BEV lipids. We then extracted the lipid fraction from the BEVs (Fig. 7a) and separated it into hydrophilic and lipophilic phases. Only the lipophilic phase effectively protected HC11 cells from oxidative stress-induced ferroptosis, promoting greater lipid droplet secretion (Fig. 7b, c), increasing extracellular and intracellular triglycerides (Fig. 7d, e), and significantly reducing ROS (Fig. 7f, g) and LPO (Fig. 7h, i) (*P* < 0.05). These effects confirm that *L. amylovorus* BEVs primarily function through their lipid components under oxidative stress.

**Fig. 8:**
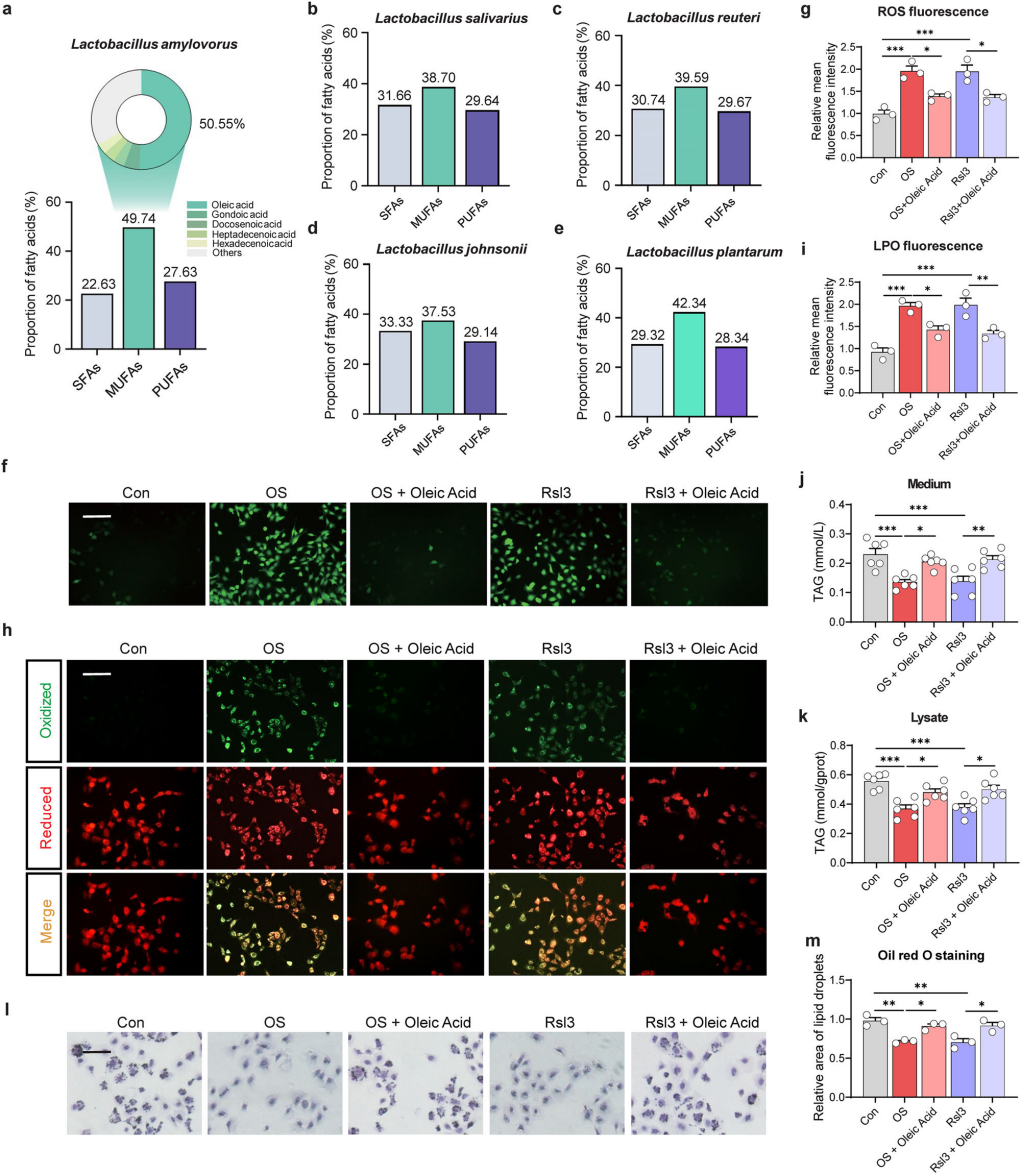
*Lactobacillus amylovorus* BEV-derived oleic acid mitigates oxidative stress-induced ferroptosis and enhances milk fat synthesis in mammary epithelial cells. **a** Content and composition of various fatty acids, including monounsaturated fatty acids, in *Lactobacillus amylovorus* extracellular vesicles. Content and composition of various fatty acids in *Lactobacillus salivarius* (**b**), *Lactobacillus reuteri* (**c**), *Lactobacillus johnsonii* (**d**), and *Lactobacillus plantarum* (**e**) BEVs. **f** Detection of intracellular reactive oxygen species (ROS) levels using ROS fluorescence assay. Scale bar: 200 μm. **g** Relative mean fluorescence intensity of ROS (*n* = 3). **h** Detection of intracellular lipid peroxidation (LPO) levels using LPO fluorescence assay. Scale bar: 200 μm. **i** Relative mean fluorescence intensity of LPO (*n* = 3). Triglyceride concentrations in the medium (**j**) and within cells (**k**) (*n* = 6). **l** Oil Red O staining of HC11. Scale bar: 100 μm. **m** Quantitative analysis of Oil Red O staining (*n* = 3). *P < 0.05; **P < 0.01; ***P < 0.001; ns not significant (*P* > 0.05).

Lipidomics analysis demonstrated that monounsaturated fatty acids (MUFAs) accounted for approximately 49.74% of the total fatty acids in the BEVs, with oleic acid representing 50.55% of the MUFAs (Fig. 8a). Compared to the other four *Lactobacillus* species, *Lactobacillus amylovorus* exhibited the highest proportion of MUFAs (Fig. 8b–e). Monounsaturated fatty acids can replace polyunsaturated fatty acids (PUFAs) in cell membranes, reducing membrane fluidity and oxidation sensitivity, thereby decreasing the risk of ferroptosis. To validate this, we added oleic acid, the most abundant MUFA in the BEVs, to mammary epithelial cells under oxidative stress and ferroptosis conditions. As expected, oleic acid treatment substantially decreased ROS (Fig. 8f, g) and LPO (Fig. 8h, i), while boosting extracellular and intracellular triglyceride content (Fig. 8j, k) and lipid droplet formation (Fig. 8l, m) (*P* < 0.05). These observations indicate that oleic acid alleviates oxidative stress-induced ferroptosis and maintains milk fat synthesis without altering ferroptosis-related gene or protein expression.

## Discussion

While numerous studies have established a close relationship between gut microbiota and host oxidative stress, the underlying mechanisms remain to be fully elucidated^13,14^. In this study, we identified oxidative stress-induced ferroptosis as a central factor leading to decreased milk lipid synthesis in the mammary glands. We observed a negative correlation between the levels of *L. amylovorus* in the maternal gut and both oxidative stress and ferroptosis in the mammary glands. Importantly, our findings demonstrate that *L. amylovorus* mitigates oxidative stress-induced ferroptosis by delivering oleic acid, a monounsaturated fatty acid, to mammary epithelial cells via BEVs. This enrichment of oleic acid enhances the cells’ resistance to ferroptosis, boosts the synthesis capacity of milk lipids in the mammary glands, and subsequently improves the growth performance of offspring under oxidative stress conditions.

*L. amylovorus* is an acidophilic lactic acid bacterium predominantly found in the intestines of humans and various animals, particularly enriched in the colon^15^. It efficiently ferments starch, produces beneficial metabolites, and maintains gut microbiota balance, thereby promoting nutrient absorption and immune function in the host^16^. *L. amylovorus* has shown potential in regulating several gut-related diseases, such as inflammatory bowel disease (IBD) and irritable bowel syndrome (IBS)^17,18^. Additionally, BEVs from *Lactobacillus* species have been implicated in regulating multiple physiological functions. For instance, *Lactobacillus johnsonii* and *Lactobacillus murinus* BEVs activate M2 polarization in intestinal epithelial cells, thereby enhancing the barrier function of the gut^19,20^. Similarly, BEVs from *Lactobacillus plantarum* influence skin health by affecting M2 macrophage polarization^21^, and BEVs from *Lactobacillus druckerii* reduce hypertrophic scar formation by inhibiting the expression of Collagen I/III and α-SMA^22^. Furthermore, *Lactobacillus rhamnosus* BEVs interact with bone physiology by promoting angiogenesis and osteogenesis, thereby reducing osteonecrosis of the femoral head^23^. These studies collectively suggest that BEVs derived from *L. amylovorus* may possess significant biological value.

In this research, we observed that BEVs secreted by *L. amylovorus* accumulate extensively in the mammary glands. We also demonstrated that these BEVs are capable of transporting substantial amounts of oleic acid into mammary epithelial cells. Although the specific mechanisms facilitating the accumulation of BEVs in the mammary glands remain unclear, our evidence strongly supports the protective role of *L. amylovorus* BEVs against oxidative stress in the mammary glands. Another key finding of our study is that oxidative stress-induced decline in mammary gland lactation is primarily due to ferroptosis. Ferroptosis is a form of programmed cell death characterized by iron-dependent lipid peroxidation, leading to the accumulation of lipid hydroperoxides containing highly unsaturated fatty acids such as arachidonic acid and linoleic acid^24,25^. A hallmark of ferroptosis includes significant mitochondrial shrinkage, increased membrane density, and the reduction or disappearance of cristae structures^26^. In this study, we observed substantial mitochondrial abnormalities and changes in key genes that exacerbate ferroptosis in mammary epithelial cells under oxidative stress. Previous research has shown that dietary monounsaturated fatty acids (MUFAs) can increase the ratio of MUFA to polyunsaturated fatty acids (PUFAs) in membrane lipids, thereby protecting cells from oxidative lipid damage^27,28^. This aligns with our findings that BEVs enriched with MUFAs from *L. amylovorus* can alleviate oxidative stress-induced ferroptosis.

In conclusion, this study reveals that ferroptosis is a critical mechanism by which oxidative stress impairs lactation performance in the mammary glands. Furthermore, *L. amylovorus* and its BEVs offer a promising therapeutic strategy to counteract oxidative stress-induced lactation decline. These insights not only enhance our understanding of the gut-mammary axis in maternal health but also pave the way for developing novel interventions to support lactation under stress conditions.

## Methods

### Animal experiments with sows

A total of 63 Landrace × Yorkshire sows (first day of lactation) with comparable backfat depths and parity numbers were selected. All sows underwent routine health checks and were found to be in good physical condition. They were housed under standard swine production conditions and had ad libitum access to feed and water. The diet was formulated according to NRC (2012) guidelines (see Supplementary Table 1). Blood samples were collected to measure the oxidative stress index (OSI), calculated as total oxidant status (TOS) divided by total antioxidant status (TAS) (Jiancheng Bioengineering Institute, Jiangsu, China). The 10 sows with the highest OSI were designated as the high oxidative stress (HOS) group, and the 10 with the lowest OSI were designated as the low oxidative stress (LOS) group. Subsequent procedures, including fecal sampling (for microbiota preservation) and colostrum collection (for analyzing milk composition and isolating mammary epithelial cells), were conducted according to these groupings.

### Animal experiments with mice

Twenty specific-pathogen-free (SPF) female ICR mice (6 weeks old) were obtained from Guangdong Sijia Jingda Biotechnology Co., Ltd. (Guangdong, China). They were randomly divided into two groups with equal distribution and housed under SPF conditions (12-h light/dark cycle, 24 °C, 55–60% humidity) with free access to food and water. In this fecal microbiota transplantation study, mice were pair-mated at 8 weeks of age, with vaginal plug confirmation establishing gestational timelines. Ten days prior to parturition, dams underwent gut microbiota depletion via broad-spectrum antibiotics (1 g/L each of neomycin, ampicillin, gentamicin, metronidazole, and 0.5 g/L vancomycin; Sigma-Aldrich, St. Louis, MO, USA) administered in drinking water. Postpartum litters were standardized to 13 pups per dam and orally gavaged daily with 200 μL of fecal suspensions (10^9^ CFU/mL) derived from either high oxidative stress sows (HOS) or low oxidative stress sows (LOS) until lactation day 14 (peak milk production). At termination, dams were euthanized via intraperitoneal sodium pentobarbital overdose (100 mg/kg), followed by cervical dislocation. Mammary gland tissue and cecal contents were aseptically collected, flash-frozen in liquid nitrogen, and stored at −80 °C for downstream omics analyses.

Four-week-old SPF female ICR mice were intraperitoneally injected daily with D-galactose (150 mg/kg; Sigma-Aldrich, St. Louis, MO, USA) for 42 consecutive days to induce oxidative stress, while control groups received equivalent volumes of sterile saline^29^. The OS + Fer-1 group was co-administered D-galactose and 10 mg/kg Ferrostatin-1 (Fer-1; Selleck Chemicals, Houston, TX, USA) daily^30^. At 10 weeks of age, mice were pair-mated, with vaginal plug detection used to establish parturition timelines. Ten days prepartum, dams underwent gut microbiota depletion via a broad-spectrum antibiotic cocktail in drinking water. Postpartum litters were standardized to 13 pups per dam, and interventions were administered from lactation day 0 to 14 as follows: LOS sow-derived fecal microbiota (oral gavage), GW4869 inhibitor (5 μM; oral gavage; Selleck Chemicals, Houston, TX, USA), *Lactobacillus amylovorus* (1 × 10^9^ CFUs; oral gavage), GW4869-pretreated *L. amylovorus*^31^ (1 × 10^9^ CFUs; oral gavage), or *L. amylovorus*-derived BEVs (2.6 × 10^10^ particles/kg body weight in 20 μL PBS; intraperitoneal injection). Control dams received sterile saline via matched routes. All groups included 8 biological replicates (n = 8 dams/group). On lactation day 14, dams were euthanized via an overdose of sodium pentobarbital (100 mg/kg), followed by cervical dislocation, and mammary tissue was aseptically collected for downstream analyses. All the animal experiments were conducted in accordance with the ethical policies and procedures approved by the Animal Care and Use Committee of South China Agricultural University (Guangzhou, China).

### Cell culture experimental design

Mouse mammary epithelial HC11 cells were maintained in DMEM/F12 medium supplemented with 10% FBS, 5 μg/mL IGF-1, 10 ng/mL EGF, 5 μg/mL ITS, and 1% penicillin-streptomycin (MCE, Monmouth Junction, NJ, USA) at 37 °C and 5% CO_2_. To induce differentiation, the cells were first cultured in a medium devoid of epidermal growth factor (EGF) for 24 h. Following this, they were transferred to DIP medium, which contained 1 μM dexamethasone, 5 μg/mL insulin, and 5 μg/mL prolactin. When cells reached ~70% confluence, they were exposed to 200 μM hydrogen peroxide (H_2_O_2_) for 12 h to induce oxidative stress^32^. In an additional group (OS + Fer-1), the cells were pretreated with 10 μM Fer-1 (Selleck Chemicals, Houston, TX, USA) for 6 h prior to H_2_O_2_ exposure^33^. Following stress induction, cells were treated for 24 h with one of the following: cell-free supernatants from various *Lactobacillus* strains (3%), supernatants of *L. amylovorus* pretreated with GW4869 (3%), *L. amylovorus*-derived BEVs (1.3 × 10^10^ particles/mL), different BEV fractions, short-chain fatty acids (SCFAs; acetate: propionate: butyrate = 65%:20%:15%, 2.5 mM), or oleic acid (50 μM). Intracellular reactive oxygen species (ROS), lipid peroxidation (LPO), and lipid accumulation were then quantified.

### Oxidative stress index (OSI) measurement

The TOS and TAS in the blood of 63 sows on the first day of lactation were measured (Jiancheng Bioengineering Institute, Jiangsu, China), and these were then used to calculate OSI (OSI = TOS/TAS). The sows were ranked based on their OSI values to categorize their oxidative stress status.

### Measurement of glutathione (GSH), malondialdehyde (MDA), and triglycerides (TG)

Blood or serum samples from sows and mice were used to measure GSH, MDA, and triglyceride (TG) levels using commercial kits (Jiancheng Bioengineering Institute, Jiangsu, China). Cultured cells were also analyzed similarly after treatment; cells were washed, lysed, and processed for GSH, MDA, or TG measurement.

### Milk composition analysis in sows

From each of the 63 sows, 50 mL of colostrum was obtained on lactation day 1 and immediately frozen at −80 °C. Fat, protein, lactose, and solids-not-fat were measured using a milk composition analyzer (Ekomilk Bond, Espoo, Finland).

### Isolation of mammary epithelial cells from sow colostrum

Colostrum was mixed with PBS in a 1:1 ratio and subjected to centrifugation at 870 × *g* for 20 min at 4 °C. The resulting pellet was resuspended in 5 mL PBS and centrifuged at 490 × *g* for 5 min. This washing procedure was repeated twice to isolate mammary epithelial cells.

### Preservation of fecal microbiota from lactating sows

Fecal samples (100 g) were taken from each of the 63 sows on lactation day 1, mixed with 300 mL sterile saline, homogenized, and filtered. The filtrate was centrifuged at 600 × *g* for 15 min, and pellets were resuspended in sterile saline with 10% glycerol. These suspensions were frozen at −80 °C for future experiments.

### 16S rRNA sequencing and data analysis

Microbial composition was analyzed via 16S rRNA next-generation sequencing (Biomarker Biotechnology, Beijing, China). Total DNA was extracted from sow fecal samples (A260/A280 ratio 1.8–2.0). Primers F (5′-ACTCCTACGGGAGGCAGCA-3′) and R (5′-GGACTACHVGGGTWTCTAAT-3′) were used to amplify the V3 + V4 regions. After purification, qPCR products were sequenced on an Illumina NovaSeq 6000 (San Diego, CA, USA).

### Quantification of bacterial load

Total DNA was extracted from fecal samples using a commercial fecal DNA extraction kit (Tiangen Biotech, Beijing, China). DNA concentration was measured with a NanoDrop 2000 spectrophotometer (Thermo Fisher Scientific, Wilmington, DE, USA) and normalized using nuclease-free water prior to RT-qPCR analysis. Bacterial load was quantified by comparing Ct values to a standard curve generated from known concentrations of bacterial DNA. Primer sequences specific to the target bacterial species are provided in Supplementary Table 3.

### Bacterial plate counting of fecal samples

Fresh fecal samples were collected from mice into sterile tubes, homogenized in sterile saline, and serially diluted up to 10^7^-fold. Aliquots of each dilution were spread onto autoclaved, non-selective plate count agar (HKM, Guangzhou, China) using disposable sterile spreaders. Plates were incubated at 37 °C for 48 h in a constant-temperature incubator, and bacterial colonies were then counted to determine viable cell numbers.

### Mitochondrial morphology analysis

On day 14 of lactation, mice were euthanized, and mammary gland tissues were collected, cut into 1 mm³ pieces, and fixed in 2.5% glutaraldehyde at 4 °C overnight. After dehydration, infiltration, and embedding, ultrathin (70 nm) sections were examined under a Talos L120C transmission electron microscope (TEM; Thermo Fisher Scientific, Eindhoven, Netherlands) to observe mitochondrial size, shape, and density. Mammary epithelial cells underwent a similar fixation and preparation process.

### Whole-mount staining

On lactation day 14, mammary gland tissues from euthanized mice were washed and fixed in Carnoy’s fixative for 12 h. Tissues were stained overnight with Carmine Alum (Sigma-Aldrich, St. Louis, MO, USA), then dehydrated in ethanol (75%, 95%, and 100%) and cleared in xylene^34^.

### Mammary tissue H&E staining

Also on lactation day 14, mammary gland tissues were harvested, washed, and fixed in 4% paraformaldehyde before paraffin embedding and sectioning. After deparaffinization in xylene and rehydration, sections were stained with hematoxylin and eosin to visualize alveolar structures, then dehydrated and mounted.

### Oil Red O staining

Mammary gland tissues were sectioned at 8 μm thickness and embedded in OCT (Sangon Biotech, Shanghai, China), then stained with filtered Oil Red O (Sangon Biotech, Shanghai, China). The nuclei were counterstained with hematoxylin (Sangon Biotech, Shanghai, China). For cell cultures, HC11 cells were fixed using 4% paraformaldehyde, stained with Oil Red O, washed, and counterstained with hematoxylin to visualize lipid droplets.

### Analysis of murine milk fat percentage

Mice were separated from pups overnight before milk collection on day 14 of lactation. Oxytocin (4 IU; MCE, Monmouth Junction, NJ, USA) was administered intraperitoneally, and milk was collected 30 min later. Samples were diluted 1:2 with PBS and centrifuged at 3000 rpm for 20 min at 4 °C. The volume of the fat layer that separated after centrifugation was measured, and the fat percentage was calculated by comparing the volume of the fat layer to the total milk volume in the capillary tube^35^.

### Purification and characterization of bacterial extracellular vesicles (BEVs)

*L. amylovorus* was cultured in MRS medium at 37 °C for 72 h. The culture supernatant was sequentially centrifuged (360 × *g*, 15 min; 3000 × *g*, 15 min; 10,000 × *g*, 15 min) to remove debris, then filtered through a 0.22 μm membrane. The filtrate was ultracentrifuged at 180,000 × *g* for 90 min at 4 °C (Beckman Optima XE-100, Beckman Coulter, Brea, CA, USA)^36^. The obtained BEV pellet was resuspended in PBS, washed, and then centrifuged at 180,000 × *g* for 90 min at 4 °C. The resulting crude BEVs were filtered through a 0.22 μm syringe filter and subsequently purified and concentrated using the ExoSure™ Exosome Isolation Kits (GeneCopoeia, Rockville, MD, USA) to isolate bacterial extracellular vesicles ranging from 30 to 150 nm. The concentration of bacterial extracellular vesicles was determined using a protein assay kit (Beyotime, Jiangsu, China). For morphological analysis, BEVs were negatively stained with phosphotungstic acid (PTA), air-dried, and visualized by transmission electron microscopy (TEM). BEV particle size and concentration were measured with a NanoSight NS300 system (Malvern Panalytical, Malvern, UK)^37^.

### Inhibition of BEVs production

*Lactobacillus amylovorus* was inoculated into autoclaved MRS medium at a concentration of 10^7^ CFUs/100 mL, with or without 10 μM GW4869, an inhibitor of extracellular vesicle secretion. Cultures were incubated at 37 °C with shaking at 120 rpm for 72 h. Following incubation, BEVs were isolated as described above, and their protein content was measured using a BCA protein assay kit (Beyotime, Jiangsu, China). Bacterial viability under GW4869 treatment was assessed by colony-forming unit (CFU) counts on MRS agar plates (HKM, Guangzhou, China) and by measuring optical density at 600 nm (OD600) using a spectrophotometer (Evolution 300, Thermo Fisher Scientific, Wilmington, DE, USA).

### Evaluation of BEVs biodistribution in tissues

On lactation day 14, Dil-labeled (MCE, Monmouth Junction, NJ, USA) BEVs were injected intraperitoneally into mice. Fluorescence signals were recorded at 1, 6, and 12 h with an IVIS Lumina III system (PerkinElmer, Waltham, MA, USA). Twelve hours post-treatment, the mice were euthanized, and the major organs (heart, liver, spleen, lungs, kidneys, and mammary glands) were harvested and analyzed to assess BEV distribution.

### Western blot

On lactation day 14, mammary glands were collected from euthanized mice, washed, minced, and lysed in RIPA buffer (Beyotime, Jiangsu, China) containing protease and phosphatase inhibitors (MCE, Monmouth Junction, NJ, USA). Homogenates were centrifuged at 12,000 × *g* for 15 min; supernatants were quantified by a BCA assay (Beyotime, Jiangsu, China). For HC11 cells, they were lysed in RIPA buffer with protease and phosphatase inhibitors. Lysates were centrifuged at 12,000 × *g* for 15 min at 4 °C, and protein concentrations were measured using the BCA assay (Beyotime, Jiangsu, China). Equal protein amounts (20–40 µg) were separated by 10–12% SDS-PAGE and transferred to a nitrocellulose membrane. The membrane was blocked with 5% non-fat milk in TBST for 1 h and incubated overnight at 4 °C with primary antibodies. After washing, the membrane was incubated with secondary antibody for 1 h, followed by chemiluminescent detection (Tanon 5200 imaging system, Tanon Science & Technology, Shanghai, China). Bands were quantified using ImageJ software. Protein levels were normalized to β-actin. All the antibody information used above can be found in Supplementary Table 4.

### RNA isolation and real-time qPCR

On lactation day 14, mammary gland tissues were dissected and washed with PBS. Total RNA was extracted (EZBioscience, Roseville, MN, USA), and for cultured cells, the cell RNA purification kit (EZBioscience, Roseville, MN, USA) was used. RNA concentration and purity were confirmed by Nanodrop (Thermo Fisher Scientific, Wilmington, DE, USA); cDNA was synthesized using 4× EZscript reverse transcription mix II (EZBioscience, Roseville, MN, USA). A 20 μL SYBR qPCR Master Mix reaction (EZBioscience, Roseville, MN, USA) was run on a QuantStudio 3 system (Applied Biosystems, Foster City, CA, USA), with β-actin as the reference gene. The relative expression levels of target genes were determined using the 2^−ΔΔCT^ method, with primer sequences provided in Supplementary Table 2.

### Analysis of cell viability, intracellular ROS, and LPO levels

HC11 cells were plated in 12-well plates and cultured until they reached ~70% confluence. Following treatments, cells were stained with 2 μM Calcein AM and 5 μM PI (MCE, Monmouth Junction, NJ, USA) to assess viability via fluorescence microscopy (NIKON ECLIPSE TiE, Nikon Instruments, Tokyo, Japan). For ROS detection, 10 μM DCFH-DA (MCE, Monmouth Junction, NJ, USA) was applied; for LPO, 2 μM BODIPY 581/591 C11 (MCE, Monmouth Junction, NJ, USA) was added. After staining, cells were visualized under a fluorescence microscope, and the intensity of the relevant fluorescence was quantified with ImageJ.

### Preparation of cell-free supernatants of *Lactobacillus*

Freeze-dried powders of *L. amylovorus*, *L. johnsonii*, *L. plantarum*, *L. reuteri*, and *L. salivarius* (BNCC, Beijing, China) were revived and adjusted to 1 × 10^5^ CFU/mL before storage at −80 °C. Each strain (2% inoculum) was cultured in 200 mL MRS at 37 °C under anaerobic conditions for 72 h. Cultures were centrifuged twice at 1,000 × *g* for 15 min (4 °C). Supernatants were filtered through 0.22 μm membranes and used for cell treatments.

### Detection of BEVs uptake by cells

After HC11 cells were treated, 5 μM Dil-labeled BEVs were introduced to the cells and incubated at 37 °C for 6 h. After incubation, the cells were washed, fixed with 4% paraformaldehyde for 30 min, and then washed again with PBS. DAPI mounting medium was then applied, and cells were viewed under an inverted fluorescence microscope. Red Dil fluorescence indicated BEV uptake, while nuclei were stained blue.

### Removal of BEV proteins and nucleic acids

BEVs isolated by ultracentrifugation at 4 °C were resuspended (1.3 mg/mL), subjected to six freeze-thaw cycles, and then treated with 30-second sonication. The samples were then treated with RNase A (1 U/μL), DNase I (2 U/μL), or proteinase K (40 μg/mL; MCE, Monmouth Junction, NJ, USA) to selectively remove RNA, DNA, or proteins, respectively. After 30-min incubation at 37 °C, reactions were terminated with PMSF (for proteinase K inactivation) or EDTA (for DNase I/RNase A inactivation) as appropriate, followed by two PBS washes prior to cellular processing.

### Lipid extraction from BEVs

The BEVs isolated by ultracentrifugation were resuspended in deionized water (1.3 mg/mL). A 200 μL aliquot of the BEVs suspension was mixed with 375 μL of chilled methanol, followed by addition of 3 volumes (1125 μL) of methyl tert-butyl ether (MTBE; Sigma-Aldrich, St. Louis, MO, USA) and vigorous shaking for 90 min. After adding 650 μL deionized water and incubating at room temperature for 15 min, the mixture was centrifuged at 10,000 × *g* for 10 min to separate the upper phase (lipophilic fraction) from the lower phase (hydrophilic fraction). The lipophilic fraction was concentrated by liquid nitrogen evaporation to obtain purified lipids for cellular treatment (20 μL/mL culture medium).

### Lipidomic analysis of BEVs

The BEVs were mixed with chloroform: methanol (2:1), followed by the addition of an equal volume of distilled water. After vortexing, the mixture was subjected to centrifugation at 3000 × *g* for 15 min, and the lower chloroform fraction was collected, dried under nitrogen, and resuspended in a suitable organic solvent (e.g., methanol or chloroform-methanol). Lipid species were profiled and fatty acid composition was determined using liquid chromatography-mass spectrometry (LC-MS; Suzhou PANOMIX Biotechnology Co., Ltd., Guangdong, China).

### Statistical analysis

Data analysis was performed using GraphPad Prism 10.1.2. For comparisons between two groups, an unpaired two-tailed Student’s t-test was employed, while one-way ANOVA with Tukey’s post hoc test was used for comparisons involving three or more groups. Results are expressed as mean ± SEM, unless stated otherwise. Statistical significance was determined at *P < 0.05, **P < 0.01, ***P < 0.001, ****P < 0.0001, and ns (not significant) for *P* > 0.05.

## Supplementary information

Supplementary Information

Supplementary File-Western Blot Bands

## Data Availability

The 16S sequencing data from this study are available in the NCBI Sequence Read Archive under the accession number PRJNA1210767. All data presented in this work are available within the article and the supplementary files. Any additional requests can be addressed to the corresponding authors.
