# Supplementary material for: Lactobacillus amylovorus extracellular vesicles mitigate mammary gland ferroptosis via the gut-mammary gland axis
Source: NPJ Biofilms Microbiomes. 2025 Jun 21;11:113. doi: 10.1038/s41522-025-00752-4 (PMC12182568; doi:10.1038/s41522-025-00752-4)
Supplement: Supplementary file 1 — Supplementary Information [file 41522_2025_752_MOESM1_ESM.pdf]

Supplementary Materials for *Lactobacillus amylovorus* Extracellular Vesicles Mitigate Mammary Gland Ferroptosis via the Gut-Mammary Gland Axis

Author Information

Author

Qianzi Zhang[1], Dongpang Chen[1], Hanting Ding[1], Qihui Li[1], Siyu Yuan[1], Haobin Li[1], Wutai Guan [1,2,3], Shihai Zhang [1,2,3]*

Affiliations

[1]Guangdong Province Key Laboratory of Animal Nutrition Control, College of Animal Science, South China Agricultural University, Guangzhou 510642, China

[2]College of Animal Science and National Engineering Research Center for Breeding Swine Industry, South China Agricultural University, Guangzhou 510642, China

[3]Guangdong Laboratory for Lingnan Modern Agriculture, South China Agricultural University, Guangzhou 510642, China

Corresponding author

*Correspondence: e-mail: zhangshihai@scau.edu.cn (S.Z.) Tel./Fax: +86-13660346296 ORCID ID: 0000-0002-5397-3354

The File Includes

Supplementary Figures

Supplementary Figure 1 to 12

Supplementary Tables

Supplementary Table 1 to 4

1. Supplementary Figures

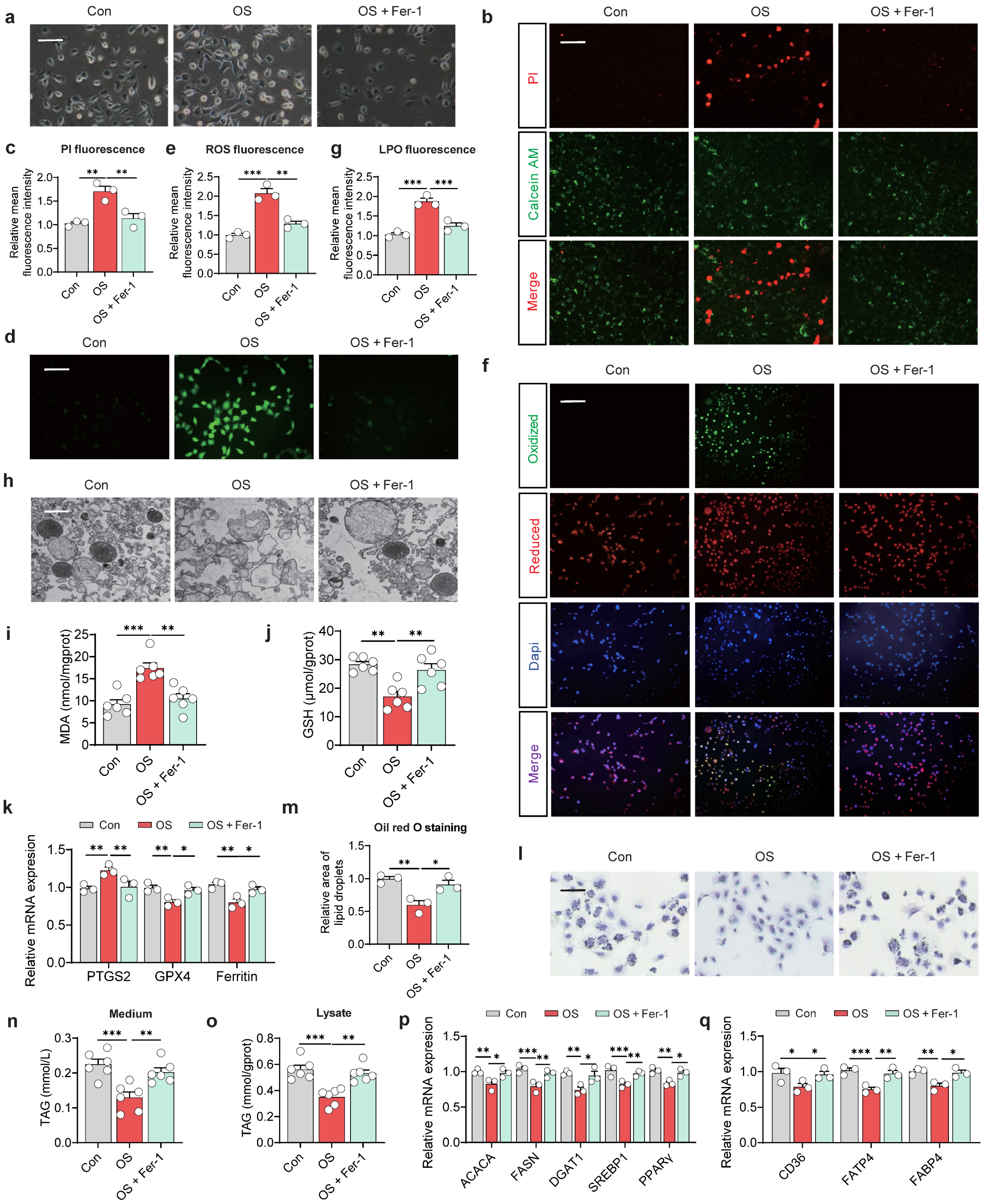

Supplementary Fig. 1 | Oxidative Stress-Induced Ferroptosis Reduces Milk Fat Synthesis in Mammary Epithelial Cells

a Cell death observed under bright-field microscopy. Scale bar: 200 μm. **b** Cell death detected using PI-Calcein AM fluorescence. Scale bar: 200 μm. **c** Relative mean fluorescence intensity of PI (n = 3). **d** Reactive oxygen species (ROS) levels measured by ROS fluorescence assay. Scale bar: 200 μm. **e** Relative mean fluorescence intensity of ROS (n = 3). **f** Intracellular lipid peroxidation (LPO) levels measured by LPO fluorescence assay. Scale bar: 500 μm. **g** Relative mean fluorescence intensity of LPO (n = 3). **h** Analysis of mitochondrial morphology. Scale bar: 500 nm. **i-j** Levels of malondialdehyde (MDA) (**i**) and glutathione (GSH) (**j**) in porcine mammary epithelial cells (pMECs) (n = 6). **k** mRNA expression of ferroptosis-related genes in pMECs (n = 3). **l** Oil Red O staining of pMECs. Scale bar: 100 μm. **m** Quantitative analysis of Oil Red O staining (n = 3). **n-o** Triglyceride concentrations in the medium (**n**) and within cells (**o**) (n = 6). **p-q** mRNA expression of milk fat synthesis-related (**p**), and transport-related (**q**) genes in pMECs (n = 3). $^{*}P < 0.05$; $^{**}P < 0.01$; $^{***}P < 0.001$; ns, not significant ($P > 0.05$).

Abbreviations:

pMECs: porcine Mammary Epithelial Cells

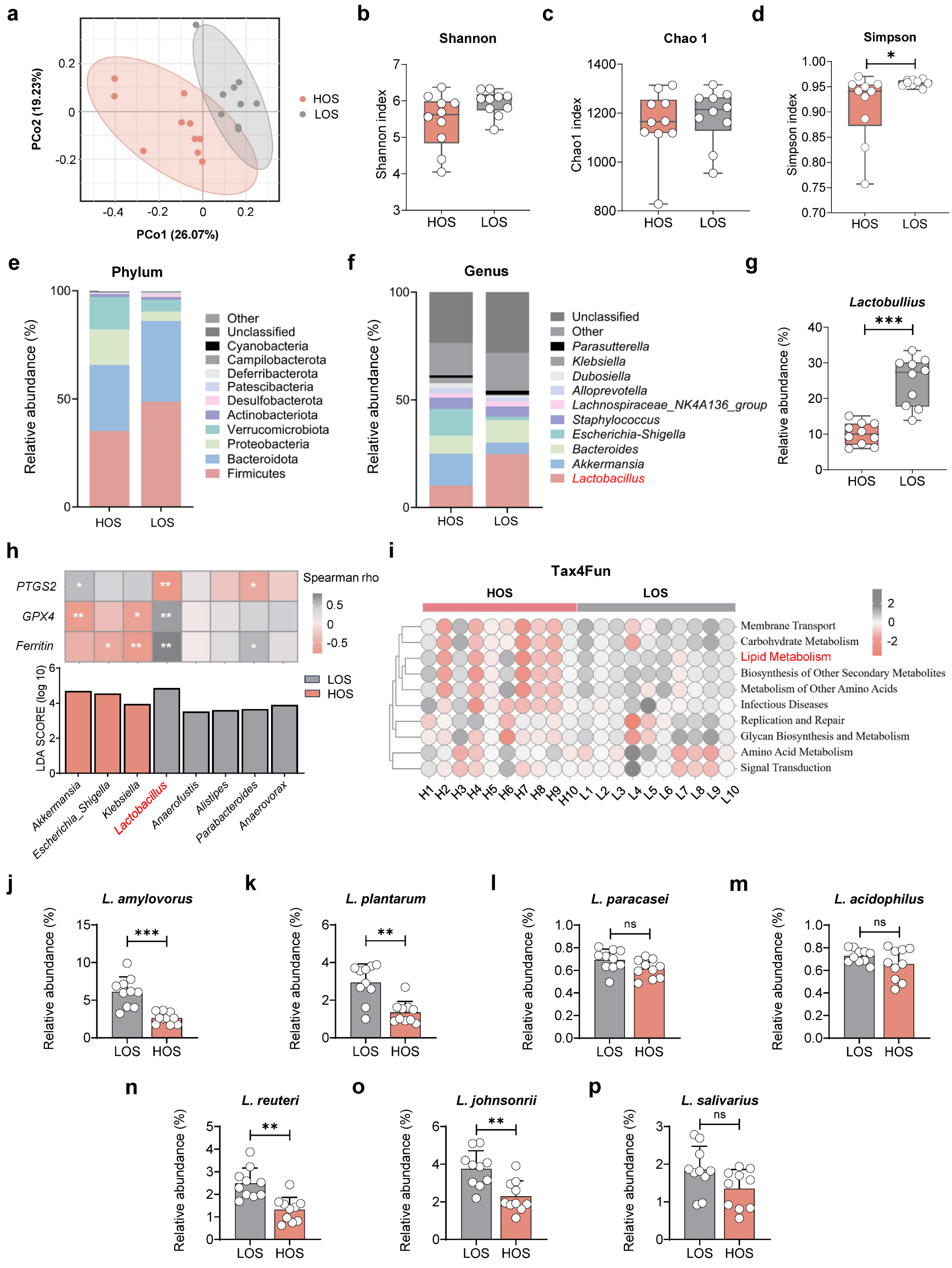
a
PCo2 (19.23%)
PCo1 (26.07%)
HOS
LOS
b
Shannon
Shannon index
c
Chao 1
Chao1 index
d
Simpson
Simpson index
e
Phylum
Relative abundance (%)
Other
Unclassified
Cyanobacteria
Campilobacterota
Deferribacterota
Patescibacteria
Desulfobacterota
Actinobacteriota
Verrucomicrobiota
Proteobacteria
Bacteroidota
Firmicutes
f
Genus
Unclassified
Other
Parasutterella
Klebsiella
Dubosiella
Alloprevotella
Lachnospiraceae_NK4A136_group
Staphylococcus
Escherichia-Shigella
Bacteroides
Akkermansia
Lactobacillus
g
Lactobullius
h
PTGS2
GPX4
Ferritin
Spearman rho
LDA SCORE (log 10)
Akkermansia
Escherichia_Shigella
Klebsiella
Lactobacillus
Anaerofustis
Alistipes
Parabacteroides
Anaerovorax
i
Tax4Fun
Membrane Transport
Carbohydrate Metabolism
Lipid Metabolism
Biosynthesis of Other Secondary Metabolites
Metabolism of Other Amino Acids
Infectious Diseases
Replication and Repair
Glycan Biosynthesis and Metabolism
Amino Acid Metabolism
Signal Transduction
j
L. amylovorus
k
L. plantarum
l
L. paracasei
m
L. acidophilus
n
L. reuteri
o
L. johnsonrii
p
L. salivarius
Relative abundance (%)

Supplementary Fig. 2 | Composition of Maternal Gut Microbiota Following Microbiota Transplantation

a Principal Coordinate Analysis of gut microbiota composition (n = 10). **b-d** Alpha diversity indices: Shannon (**b**), Chao1 (**c**), and Simpson (**d**) (n = 10). **e** Relative abundance of the top 10 bacterial phyla in fecal microbiota. **f** Relative abundance of the top 10 bacterial genera. **g** Comparison of *Lactobacillus* abundance between groups (n = 10). **h** Linear Discriminant Analysis identifying differentially abundant bacteria between HOS and LOS groups (LDA > 3.5, $P < 0.05$) and Spearman's correlations between selected bacteria and ferroptosis genes. **i** Functional prediction of microbiota using Tax4Fun analysis (n = 10). **j-p** Relative abundance of dominant *Lactobacillus* species in the intestinal microbiota of mice (n = 3). *$P < 0.05$; **$P < 0.01$; ***$P < 0.001$; ns, not significant ($P > 0.05$).

Abbreviations:

LOS: Mice under low oxidative stress

HOS: Mice under high oxidative stress

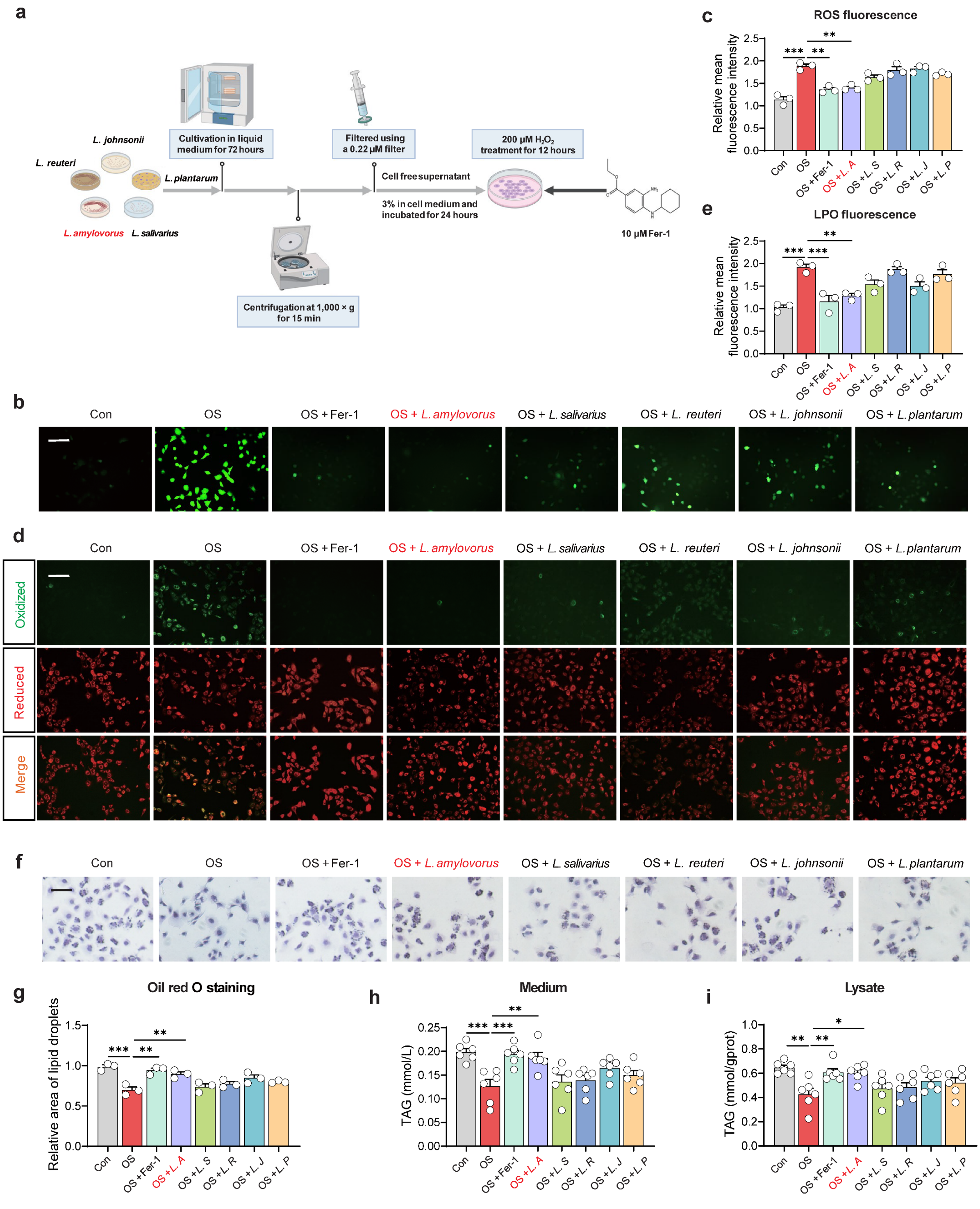
a
L. johnsonii
L. reuteri
L. plantarum
L. amylovorus
L. salivarius
Cultivation in liquid medium for 72 hours
Centrifugation at 1,000 × g for 15 min
Filtered using a 0.22 μM filter
Cell free supernatant
3% in cell medium and incubated for 24 hours
200 μM H_2O_2 treatment for 12 hours
10 μM Fer-1
b
Con
OS
OS + Fer-1
OS + L. amylovorus
OS + L. salivarius
OS + L. reuteri
OS + L. johnsonii
OS + L. plantarum
c
ROS fluorescence
Relative mean fluorescence intensity
d
Oxidized
Reduced
Merge
e
LPO fluorescence
f
g
Oil red O staining
Relative area of lipid droplets
h
Medium
TAG (mmol/L)
i
Lysate
TAG (mmol/gprot)
Con
OS
OS + Fer-1
OS + L. A
OS + L. S
OS + L. R
OS + L. J
OS + L. P

Supplementary Fig. 3 | Identification of Critical *Lactobacillus* Strains in Mitigating Oxidative Stress-Induced Ferroptosis

a Method for preparing cell-free supernatant from *Lactobacillus* strains, created with BioRender. **b** Detection of intracellular reactive oxygen species (ROS) levels using ROS fluorescence assay. Scale bar: 200 μm. **c** Relative mean fluorescence intensity of ROS (n = 3). **d** Detection of intracellular lipid peroxidation (LPO) levels using LPO fluorescence assay. Scale bar: 200 μm. **e** Relative mean fluorescence intensity of LPO (n = 3). **f** Oil Red O staining of mouse mammary epithelial cells (HC11). Scale bar: 100 μm. **g** Quantitative analysis of Oil Red O staining (n = 3). **h-i** Triglyceride concentrations in the medium (**h**) and within cells (**i**) (n = 6). *$P < 0.05$; **$P < 0.01$; ***$P < 0.001$; ns, not significant ($P > 0.05$).

Abbreviations:

L. A: *Lactobacillus amylovorus* cell-free supernatant

L. S: *Lactobacillus salivarius* cell-free supernatant

L. R: *Lactobacillus reuteri* cell-free supernatant

L. J: *Lactobacillus johnsonii* cell-free supernatant

L. P: *Lactobacillus plantarum* cell-free supernatant

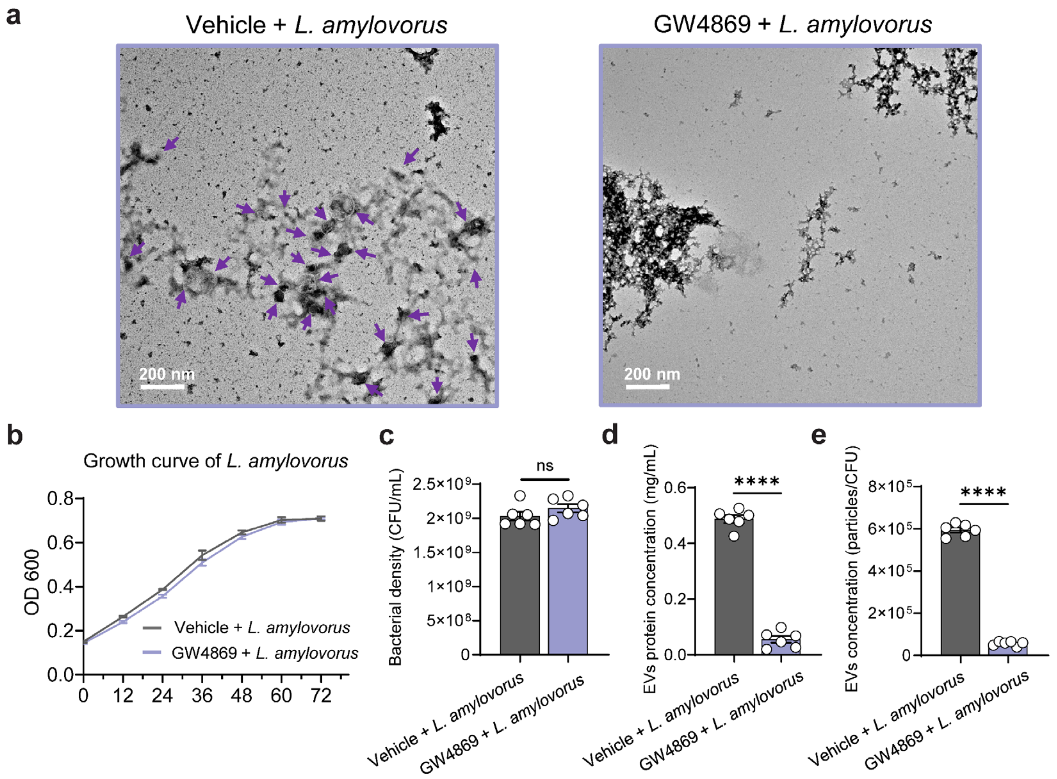

Supplementary Fig. 4 | GW4869 Inhibits the Secretion of Bacterial Extracellular Vesicles (BEVs) by *L. amylovorus*

a Transmission electron microscopy images showing BEVs released by *L. amylovorus* under control conditions (left) and following GW4869 treatment (right). Arrows indicate extracellular vesicles, which appear as single-membrane, spherical or elliptical structures (50–150 nm in diameter). A notable reduction in vesicle presence is observed after GW4869 exposure. **b** Growth curves of *L. amylovorus* treated with vehicle (DMSO) or GW4869, measured by OD600 over time (n = 3). **c** Viability of *L. amylovorus* assessed by colony-forming units (n = 6). **d** Total protein content of purified BEVs (n = 6). **e** BEV concentration normalized to particles per CFU (n = 6). $*P < 0.05$; $**P < 0.01$; $***P < 0.001$, $****P < 0.0001$; ns, not significant ($P > 0.05$).

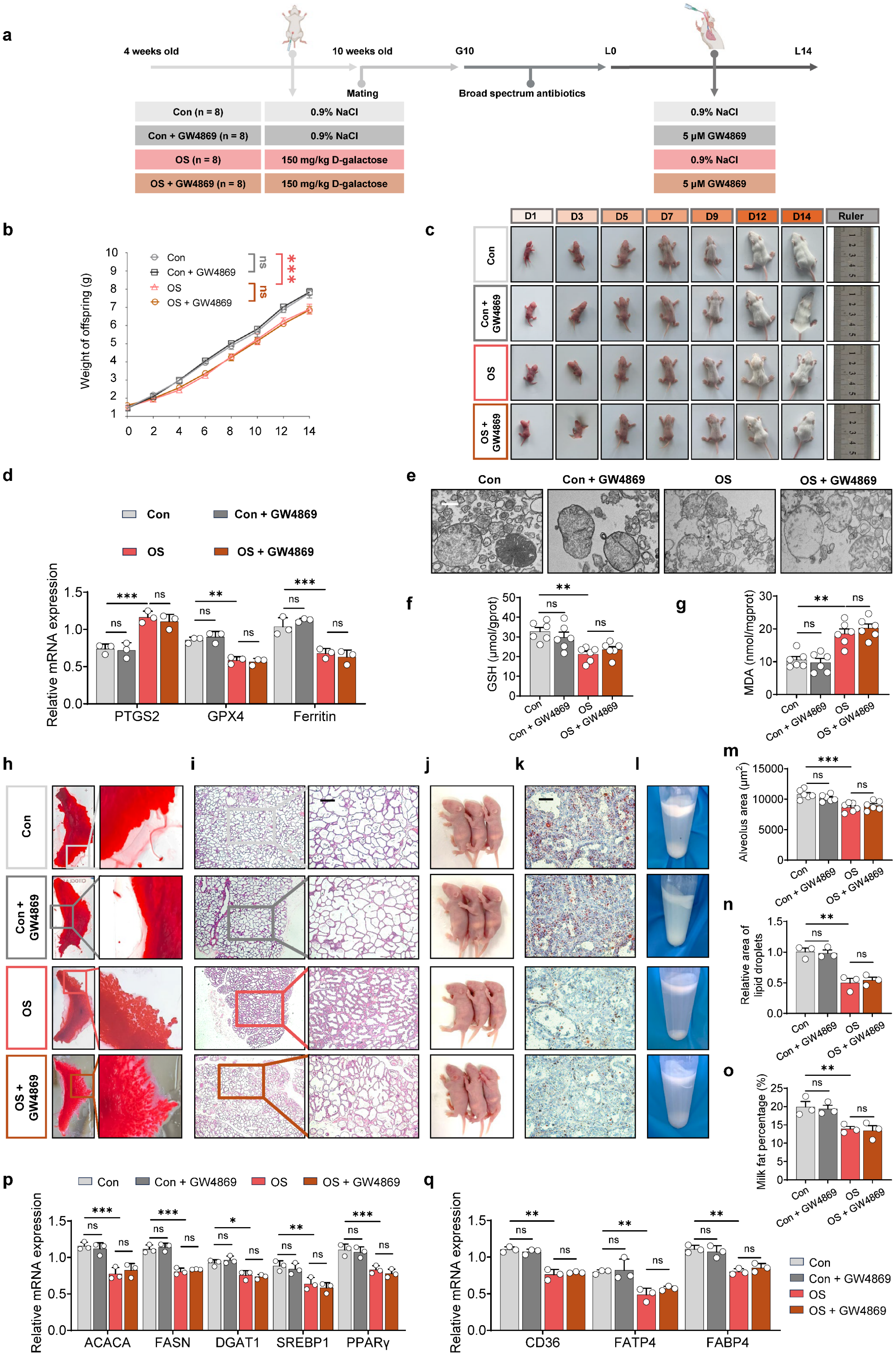
a
4 weeks old
10 weeks old
Mating
G10
Broad spectrum antibiotics
L0
L14
Con (n = 8)
0.9% NaCl
Con + GW4869 (n = 8)
0.9% NaCl
OS (n = 8)
150 mg/kg D-galactose
OS + GW4869 (n = 8)
150 mg/kg D-galactose
0.9% NaCl
5 μM GW4869
0.9% NaCl
5 μM GW4869
b
Weight of offspring (g)
Con
Con + GW4869
OS
OS + GW4869
ns
***
ns
c
D1
D3
D5
D7
D9
D12
D14
Ruler
Con
Con + GW4869
OS
OS + GW4869
d
Con
Con + GW4869
OS
OS + GW4869
Relative mRNA expression
PTGS2
GPX4
Ferritin
e
Con
Con + GW4869
OS
OS + GW4869
f
GSH (μmol/gprot)
g
MDA (nmol/mgprot)
h
i
j
k
l
m
Alveolus area (μm²)
n
Relative area of lipid droplets
o
Milk fat percentage (%)
p
Relative mRNA expression
ACACA
FASN
DGAT1
SREBP1
PPARγ
q
Relative mRNA expression
CD36
FATP4
FABP4

Supplementary Fig. 5 | The Effect of GW4869 Inhibitor Treatment Alone on Ferroptosis and Lactation Performance in Mice

a Experimental design for the effect of GW4869 treatment alone on mice, created with BioRender. **b** Body weights of pups (n = 6). Litter sizes were adjusted to 13 for each mother on L0. **c** Offspring development metrics. **d** mRNA expression of ferroptosis-related genes in mammary gland tissue of mice (n = 3). **e** Mitochondrial morphology analysis. Scale bar: 500 nm. **f-g** Levels of glutathione (GSH) (**f**), malondialdehyde (MDA) (**g**) in mouse blood (n = 6). **h** Carmine-stained whole-mount analysis of mammary glands on lactation day 14. **i** Histological sections of mammary glands stained with hematoxylin and eosin on lactation day 14. Scale bar: 200 μm. **j** Size of milk clots in the stomachs of mouse pups (n = 3). **k** Oil Red O staining of milk fat in mammary gland sections. Scale bar: 200 μm. **l** Milk fat layer after centrifugation. **m-o** Quantitative analysis of alveolus area (**m**), lipid droplet area (**n**), and milk fat percentage (**o**) (n = 3). **p** mRNA expression of milk fat synthesis-related genes in mammary gland tissue of mice (n = 3). **q** mRNA expression of milk fat transport-related genes in mammary gland tissue of mice (n = 3). *$P < 0.05$; **$P < 0.01$; ***$P < 0.001$; ns, not significant ($P > 0.05$).

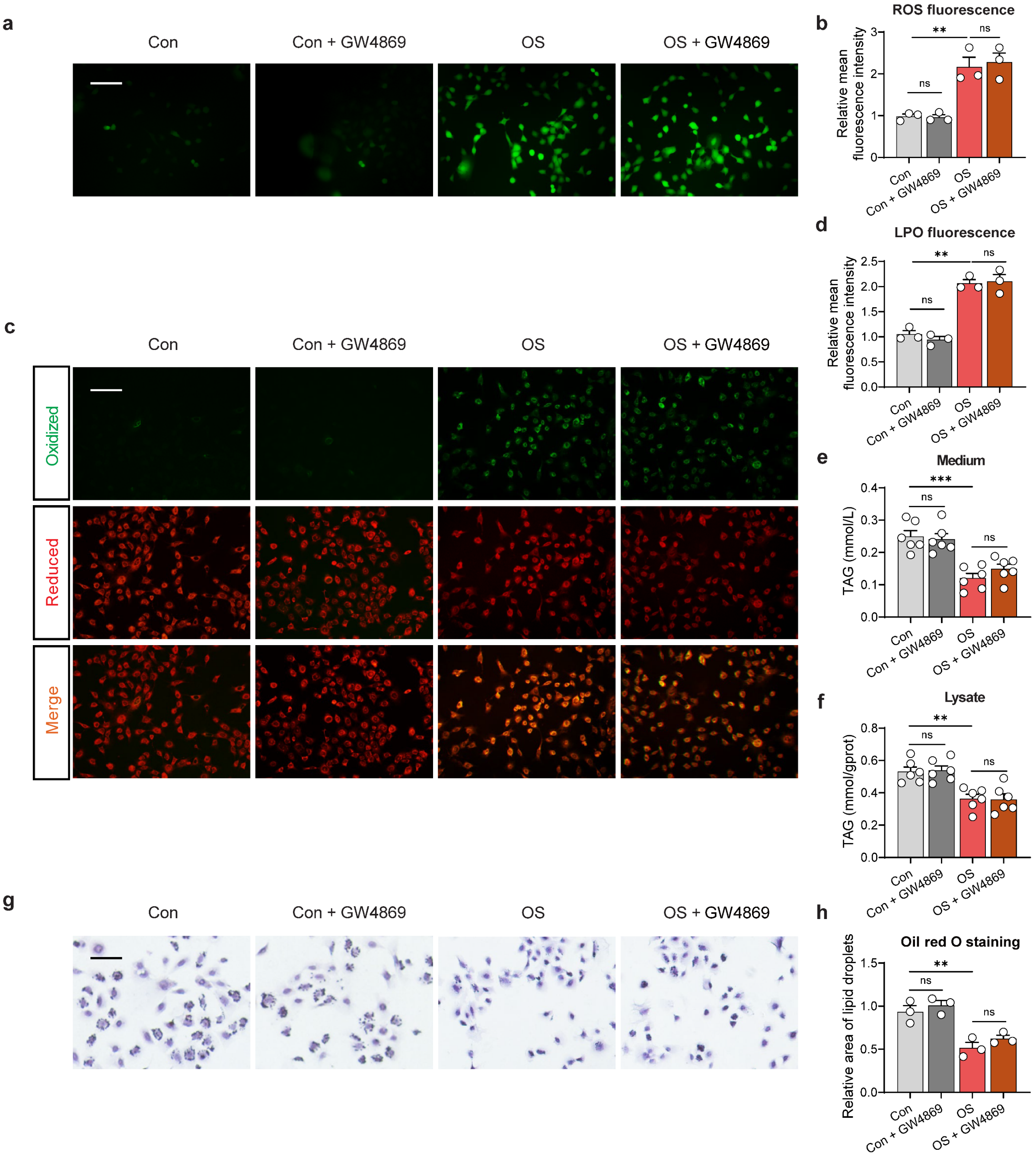
a
Con
Con + GW4869
OS
OS + GW4869
b
ROS fluorescence
Relative mean fluorescence intensity
**
ns
ns
c
Oxidized
Reduced
Merge
d
LPO fluorescence
Relative mean fluorescence intensity
e
Medium
TAG (mmol/L)
***
f
Lysate
TAG (mmol/gprot)
g
h
Oil red O staining
Relative area of lipid droplets

Supplementary Fig. 6 | The Effect of GW4869 Inhibitor Treatment Alone on Ferroptosis and Milk Fat Synthesis in HC11 Cells

a Detection of intracellular reactive oxygen species (ROS) levels using ROS fluorescence assay. Scale bar: 200 μm. **b** Relative mean fluorescence intensity of ROS (n = 3). **c** Detection of intracellular lipid peroxidation (LPO) levels using LPO fluorescence assay. Scale bar: 200 μm. **d** Relative mean fluorescence intensity of LPO (n = 3). **e-f** Triglyceride concentrations in the medium (**e**) and within cells (**f**) (n = 6). **g** Oil Red O staining of HC11. Scale bar: 100 μm. **h** Quantitative analysis of Oil Red O staining (n = 3). *$P < 0.05$; **$P < 0.01$; ***$P < 0.001$; ns, not significant ($P > 0.05$).

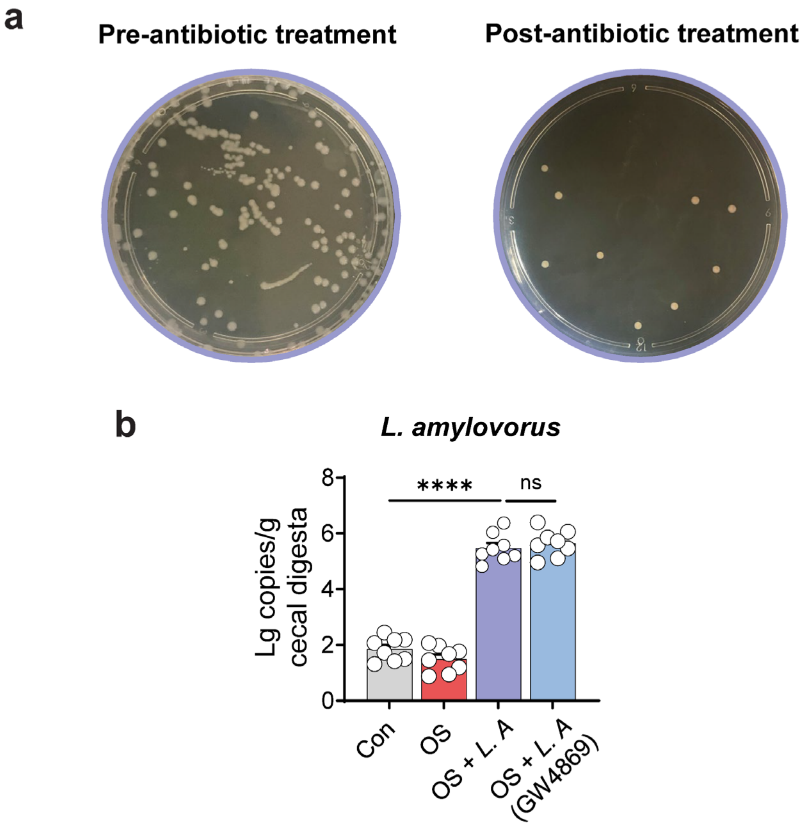

Supplementary Fig. 7 | Total bacterial load and *L. amylovorus* colonization in the intestines of dams

a Total bacterial load in dam feces before and after antibiotic treatment. **b** Copy number of *L. amylovorus* in the cecum on day 14 of lactation (n = 8).

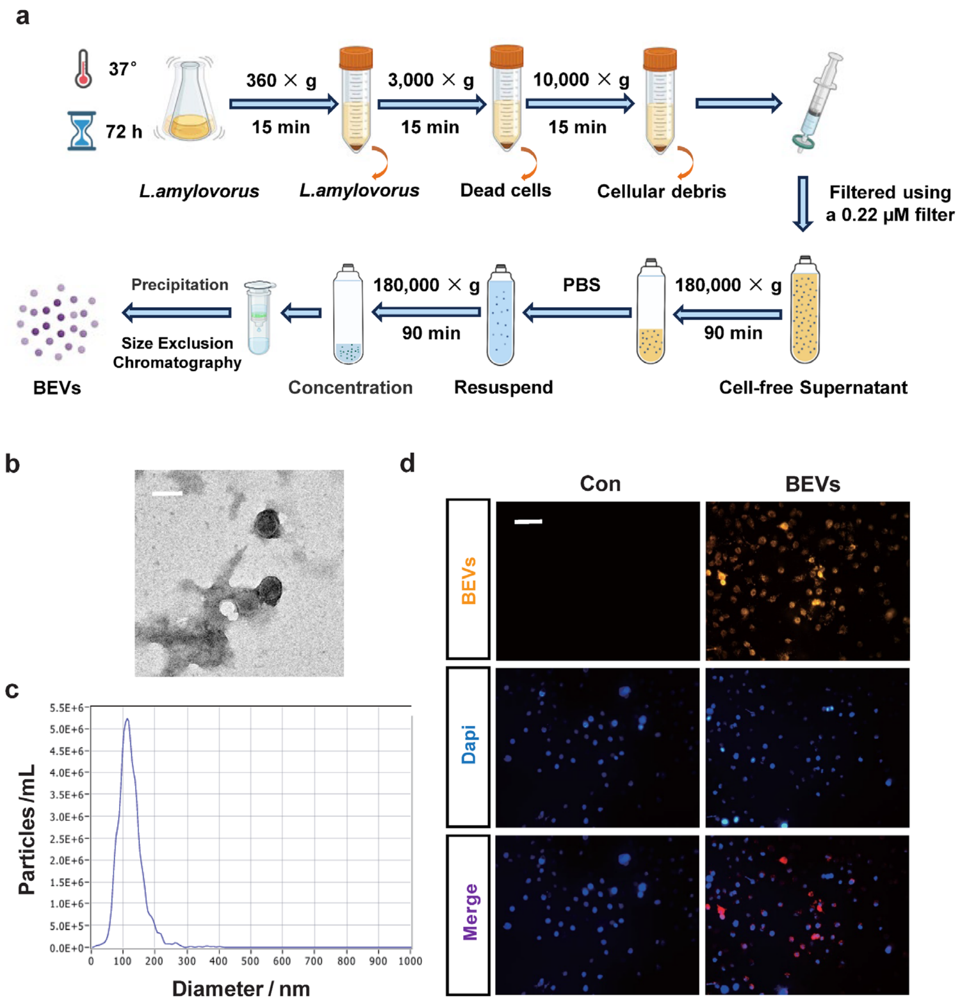

Supplementary Fig. 8 | Isolation of *Lactobacillus amylovorus* BEVs

a Ultracentrifugation process used to separate bacterial extracellular vesicles from *Lactobacillus amylovorus* culture medium, created with BioRender. **b** Transmission electron microscopy images of *Lactobacillus amylovorus* BEVs. Scale bars: 100 nm. **c** Nanoparticle tracking analysis of BEVs isolated from *Lactobacillus amylovorus*. **d** Fluorescence imaging demonstrating the uptake of BEVs by cells. Scale bar: 200 μm.

Abbreviations:

BEVs: Bacterial extracellular vesicles isolated from *Lactobacillus amylovorus*

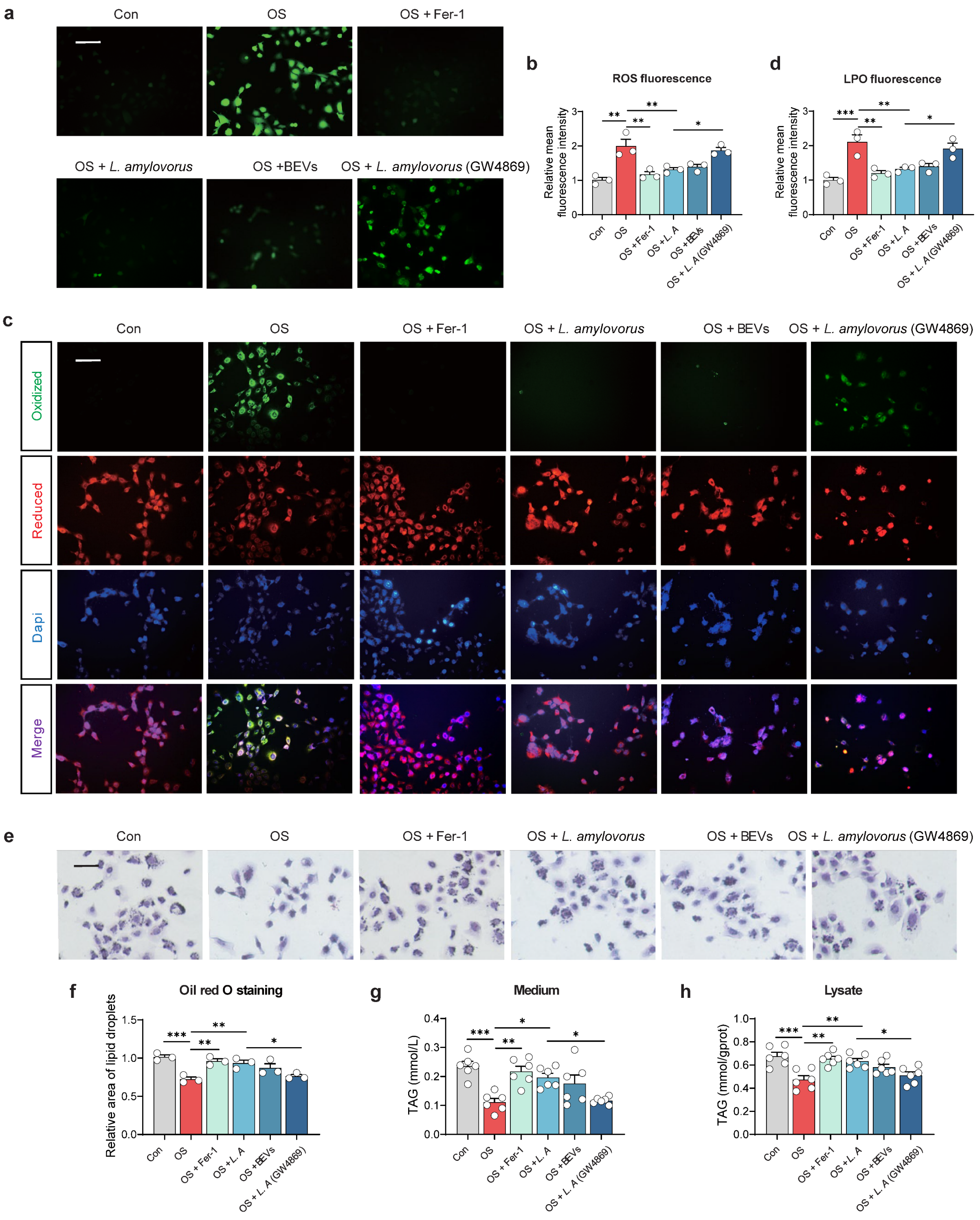
a
Con
OS
OS + Fer-1
OS + L. amylovorus
OS +BEVs
OS + L. amylovorus (GW4869)
b
ROS fluorescence
Relative mean fluorescence intensity
Con
OS
OS +Fer-1
OS +L. A
OS +BEVs
OS + L. A (GW4869)
d
LPO fluorescence
Relative mean fluorescence intensity
c
Oxidized
Reduced
Dapi
Merge
OS + BEVs
e
f
Oil red O staining
Relative area of lipid droplets
g
Medium
TAG (mmol/L)
h
Lysate
TAG (mmol/gprot)

Supplementary Fig. 9 | Partial mitigation of oxidative stress-induced ferroptosis and preservation of milk fat synthesis in mammary epithelial cells by *Lactobacillus amylovorus* via BEVs

a Detection of intracellular reactive oxygen species (ROS) levels using ROS fluorescence assay. Scale bar: 200 μm. **b** Relative mean fluorescence intensity of ROS (n = 3). **c** Detection of intracellular lipid peroxidation (LPO) levels using LPO fluorescence assay. Scale bar: 200 μm. **d** Relative mean fluorescence intensity of LPO (n = 3). **e** Oil Red O staining of HC11. Scale bar: 100 μm. **f** Quantitative analysis of Oil Red O staining (n = 3). **g-h** Triglyceride concentrations in the medium (**g**) and within cells (**h**) (n = 6). *$P < 0.05$; **$P < 0.01$; ***$P < 0.001$; ns, not significant ($P > 0.05$).

Abbreviations:

L. A: *Lactobacillus amylovorus* cell-free supernatant

L. A (GW4869): *Lactobacillus amylovorus* cell-free supernatant with GW4869- mediated extracellular vesicle depletion

BEVs: Bacterial extracellular vesicles isolated from *Lactobacillus amylovorus*

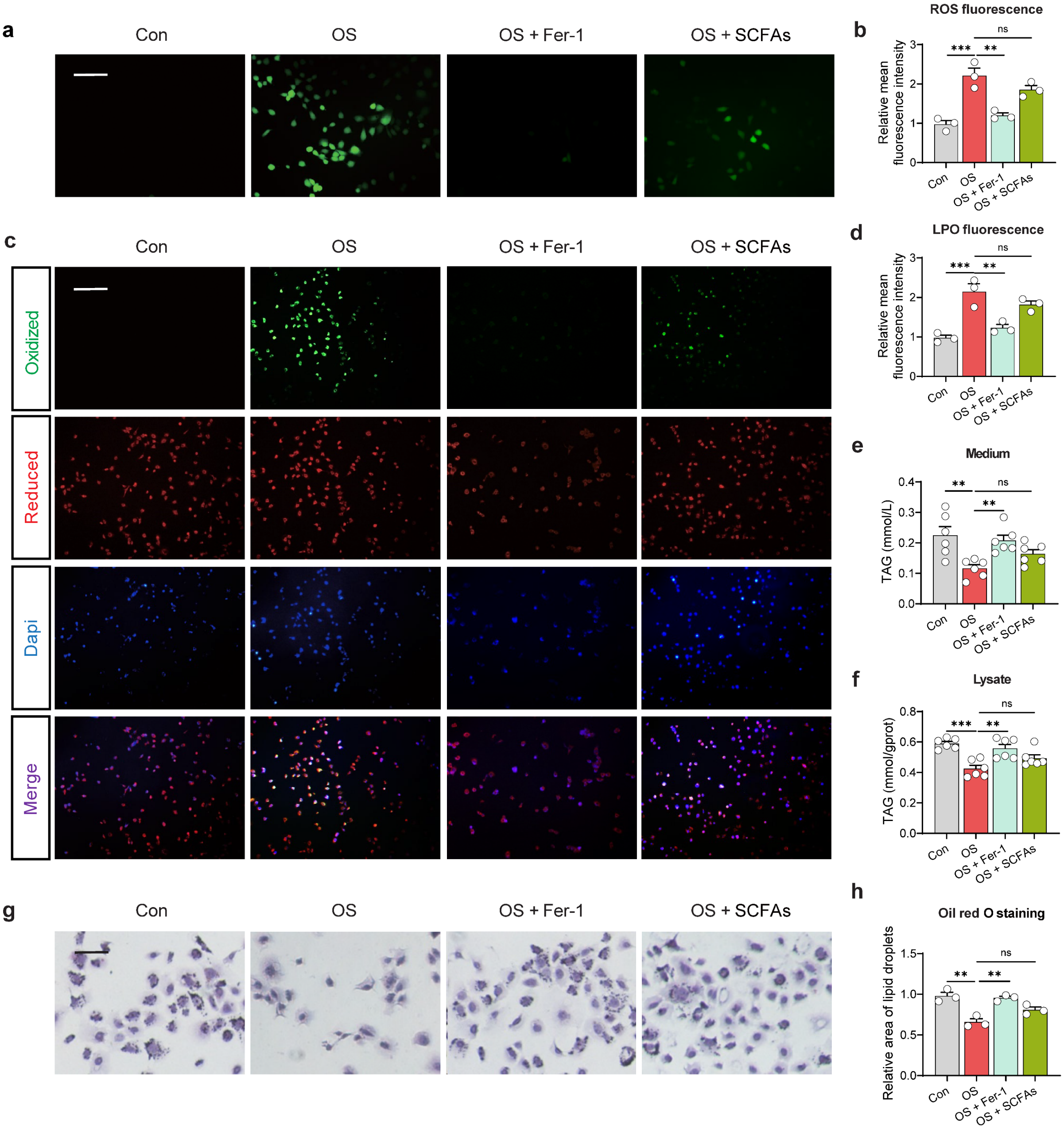
a
Con
OS
OS + Fer-1
OS + SCFAs
b
ROS fluorescence
Relative mean fluorescence intensity
*** ** ns
c
Oxidized
Reduced
Dapi
Merge
d
LPO fluorescence
Relative mean fluorescence intensity
*** ** ns
e
Medium
TAG (mmol/L)
** ** ns
f
Lysate
TAG (mmol/gprot)
*** ** ns
g
h
Oil red O staining
Relative area of lipid droplets
** ** ns

Supplementary Fig. 10 | Partial reduction of oxidative stress-induced ferroptosis and decreases in milk fat synthesis in mammary epithelial cells by *Lactobacillus amylovorus* via short-chain fatty acids

a Detection of intracellular reactive oxygen species (ROS) levels using ROS fluorescence assay. Scale bar: 200 μm. **b** Relative mean fluorescence intensity of ROS (n = 3). **c** Detection of intracellular lipid peroxidation (LPO) levels using LPO fluorescence assay. Scale bar: 500 μm. **d** Relative mean fluorescence intensity of LPO (n = 3). **e-f** Triglyceride concentrations in the medium (**e**) and within cells (**f**) (n = 6). **g** Oil Red O staining of HC11. Scale bar: 100 μm. **h** Quantitative analysis of Oil Red O staining (n = 3). $^{*}P < 0.05$; $^{**}P < 0.01$; $^{***}P < 0.001$; ns, not significant ($P > 0.05$).

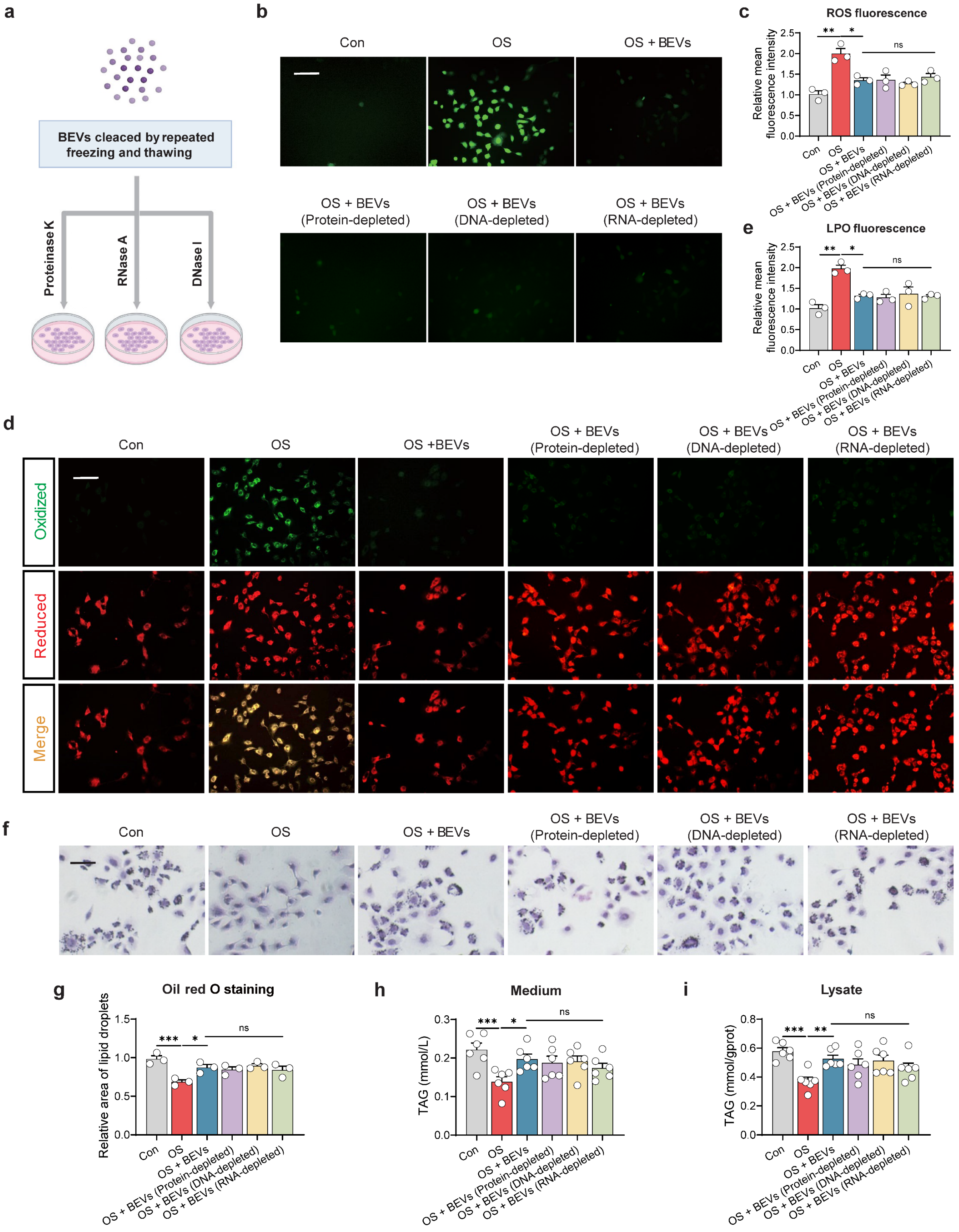

a
BEVs cleaced by repeated freezing and thawing
Proteinase K
RNase A
DNase I
b
Con
OS
OS + BEVs
OS + BEVs (Protein-depleted)
OS + BEVs (DNA-depleted)
OS + BEVs (RNA-depleted)
c
ROS fluorescence
Relative mean fluorescence intensity
** * ns
e
LPO fluorescence
Relative mean fluorescence intensity
** * ns
d
Con
OS
OS +BEVs
OS + BEVs (Protein-depleted)
OS + BEVs (DNA-depleted)
OS + BEVs (RNA-depleted)
Oxidized
Reduced
Merge
f
Con
OS
OS + BEVs
OS + BEVs (Protein-depleted)
OS + BEVs (DNA-depleted)
OS + BEVs (RNA-depleted)
g
Oil red O staining
Relative area of lipid droplets
*** * ns
h
Medium
TAG (mmol/L)
*** * ns
i
Lysate
TAG (mmol/gprot)
*** ** ns

Supplementary Fig. 11 | Alleviation of oxidative stress-induced ferroptosis and enhancement of milk fat synthesis by *Lactobacillus amylovorus* bacterial extracellular vesicles, independent of proteins, DNA, and RNA

a Experimental design for evaluating the protective effects of bacterial extracellular vesicles depleted of DNA, RNA, or proteins on oxidative stress in HC11 mouse mammary epithelial cells, created with BioRender. **b** Detection of intracellular reactive oxygen species (ROS) levels using ROS fluorescence assay. Scale bar: 200 μm. **c** Relative mean fluorescence intensity of ROS (n = 3). **d** Detection of intracellular lipid peroxidation (LPO) levels using LPO fluorescence assay. Scale bar: 200 μm. **e** Relative mean fluorescence intensity of LPO (n = 3). **f** Oil Red O staining of HC11. Scale bar: 100 μm. **g** Quantitative analysis of Oil Red O staining (n = 3). **h-i** Triglyceride concentrations in the medium (**h**) and within cells (**i**) (n = 6). *$P < 0.05$; **$P < 0.01$; ***$P < 0.001$; ns, not significant ($P > 0.05$).

Abbreviations:

BEVs: Bacterial extracellular vesicles isolated from *Lactobacillus amylovorus*.

2. Supplementary Tables

Supplementary Table 1. Composition and nutritional content of the sow diet

**Ingredient %**	**Gestation**
Corn	65.24
Wheat bran	8
Soybean meal	17.9
Soybean oil	3.3
Dicalcium phosphate	1.31
Limestone	0.97
L-Lysine hydrochloride	0.71
Threonine	0.06
Tryptophan	0.01
Premix*	2.5
Total	100
**Analyzed nutrient composition**	
Dry matter, %	87.64
Digestible energy, Mcal/kg	3.38
Metabolizable energy, Mcal/kg	3.18
Net energy, Mcal/kg	2.5
Crude protein, %	15.79
Crude fat, %	6.18
Neutral detergent fiber, %	11.12
Acid detergent fiber, %	3.9
Calcium, %	0.75
Total phosphorus, %	0.63
Non-phytate phosphorus, %	0.35
NaCl	0.3
Lysine, %	1.26

Methionine, %	0.25
Threonine, %	0.62
Tryptophan, %	0.18
Calcium, %	0.73

Supplementary Table 2. Primers for Real-Time qPCR of Target Genes

Gene	Direction	Primer sequence (5′→ 3′)
HC11 cells		
FASN	Forward	AGCACTGCCTTCGGTTCAGTC
	Reverse	AAGAGCTGTGGAGGCCACTTG
ACACA	Forward	GAAGTCAGAGCCACGGCACA
	Reverse	GGCAATCTCAGTTCAAGCCAGTC
DGAT1	Forward	CAGCTGTGGCCTTACTGGTTGA
	Reverse	CGGCACCACAGGTTGACATC
SREBP1	Forward	CGCTCTTCCATCAACGACAA
	Reverse	ATGTCTTCGAAAGTGCAATCC
FABP4	Forward	CATCCGGTCAGAGAGTACTTTT
	Reverse	TAGGGTTATGATGCTCTTCACC
PPARγ	Forward	TGACTTGAACGACCAAGTAACTC
	Reverse	CTAGTACAAGTCCTTGTAGATCTC
FATP4	Forward	CCCTGTCTACCTGCTGTTTGTC
	Reverse	CCAGCAGAGAGACAGAAGAAGG
CD36	Forward	CCTGGGAGTTGGCGAGAAA
	Reverse	CGATCACAGCCCATTCTCCT
PTGS2	Forward	CAGCTGTGGCCTTACTGGTTGA
	Reverse	CGGCACCACAGGTTGACATC
Ferritin	Forward	TGCCATCAACCGCCAGATCAAC
	Reverse	AGTTCTTCAGAGCCACATCATCTCG
GPX4	Forward	ATAAGAACGGCTGCGTGGTGAAG
	Reverse	TAGAGATAGCACGGCAGGTCCTTC
β-actin	Forward	CCACCATGTACCCAGGCATT
	Reverse	CGGACTCATCGTACTCCTGC
pMECs		

FASN	Forward	ATGGCCTCGGAGCTGTTCGA
	Reverse	CCACCTTGATGTCGTAGAGC
ACACA	Forward	AGAAGAGTGTGGCTGTGGAA
	Reverse	GCAGCTCTTTCTCCAGCATT
DGAT1	Forward	GGAAGCCAGTGAAGAGGAGA
	Reverse	GTGGAAGAGGAAGCCGATG
SREBP1	Forward	AGCTGACCTGAAAGCCGAGT
	Reverse	AGCAGATCCAGGTCAACAGC
FABP4	Forward	TCAACTTAGATGAAGTCGC
	Reverse	TCGACTTTCCATCCCACTT
PPARγ	Forward	CCTATTGACCCAGAAAGCGATT
	Reverse	CATTACGGAGAGATCCACGGA
FATP4	Forward	CTGGAAGCCCTGTCCTCTTC
	Reverse	ATGTCAGCAATGGCACAGAG
CD36	Forward	TGTGTCCTCTGATATCTGCC
	Reverse	ACACACAGCAAGCATTCACA
PTGS2	Forward	GTCTGGTGCCTGGTCTGATG
	Reverse	TCTGAGTGTCTTTGACTGTGGGAG
Ferritin	Forward	GGCTTCAACCTCAAGAAACT
	Reverse	CTTGAAGTAGCCGTAGATGG
GPX4	Forward	AGGCAAGACCGAAGTAAACTAC
	Reverse	CCGAACTGGTTACACGGGAAT
β-actin	Forward	CCAAGGCCAACCGTGAGAAGA
	Reverse	GCCAGAGGCGTACAGGGATA

Supplementary Table 3. Primers for Real-Time qPCR of *Lactobacillus* Species

Species	Direction	Primer sequence (5′→ 3′)
L. johnsonii	Forward	AGACCCAAAGGCGCTTATAGA
	Reverse	TGTAAGTTCAGAAAAATGTATCCCG
L. salivarius	Forward	CGCGTGCTTAACACATGCAA
	Reverse	TCAEAACTGAGACTGGCTTGCT
L. amylovorus	Forward	AGCGTAGGCGGTTTACTTG
	Reverse	CCATTGTGGAAGATTCCC
L. plantarum	Forward	GGTGGCTGGTTGAGTGACT
	Reverse	GCCGATACCGTTGGAAATTA
L. reuteri	Forward	CAGACAATCTTTGATTGTTTAG
	Reverse	GCTTGTTGGTTTGGGCTCTTC
L. acidophilus	Forward	CTTCGGTGATGACGTTGGGA
	Reverse	CCAATGTGGCCGATCAGTCT
L. paracasei	Forward	TACTAAAGAAATTAGCTTTTGT
	Reverse	AGTAATGTCTGCATCCTCCA
Eubacteria	Forward	ACTCCTACGGGAGGCAGCAG
	Reverse	ATTACCGCGGCTGCTGG

Supplementary Table 4. Antibody information

Antibody	Cat	Company	Dilution ratio
FASN	Abcam	ab99539	1000
ACACA	Abcam	ab72046	1000
FABP4	Abcam	ab92501	1000
DGAT1	Abcam	ab181180	1000
SREBP1	Abcam	ab3259	1000
PPARγ	Abcam	ab178860	1000
CD36	Abcam	ab133625	1000
FATP4	Abcam	ab47616	1000
PTGS2	CST	37843	1000
GPX4	Tanon	381958	100
FTH	Tanon	381204	100
β-actin	Abcam	ab8226	2000
Goat Anti-Rabbit IgG	Zenbio	511203	5000
Goat Anti-Mouse IgG	Zenbio	511103	5000

NOTE: Please save this file locally before filling in the table, DO NOT work on the file within your internet browser as changes will not be saved. Adobe Acrobat Reader (available free here) is recommended for completion.

The ARRIVE guidelines 2.0: author checklist

The ARRIVE Essential 10

These items are the basic minimum to include in a manuscript. Without this information, readers and reviewers cannot assess the reliability of the findings.

Item		Recommendation	Section/line number, or reason for not reporting
**Study design**	1	For each experiment, provide brief details of study design including:	
		a. The groups being compared, including control groups. If no control group has been used, the rationale should be stated.	lines 382-386, 397-399, 403-414 Fig. 1b, 3a, 4a, 5a, 6a, and Supplementary Fig. 5a
		b. The experimental unit (e.g. a single animal, litter, or cage of animals).	lines 384-385, 391, 414
**Sample size**	2	a. Specify the exact number of experimental units allocated to each group, and the total number in each experiment. Also indicate the total number of animals used.	lines 378, 384-386, 390-392, 415 Fig. 1b, 3a, 4a, 5a, 6a, and Supplementary Fig. 5a
		b. Explain how the sample size was decided. Provide details of any *a priori* sample size calculation, if done.	lines 384-385
**Inclusion and exclusion criteria**	3	a. Describe any criteria used for including and excluding animals (or experimental units) during the experiment, and data points during the analysis. Specify if these criteria were established *a priori*. If no criteria were set, state this explicitly.	lines 382-386, 390, 397, 403, 409
		b. For each experimental group, report any animals, experimental units or data points not included in the analysis and explain why. If there were no exclusions, state so.	N/A
		c. For each analysis, report the exact value of *n* in each experimental group.	Every figure legends
**Randomisation**	4	a. State whether randomisation was used to allocate experimental units to control and treatment groups. If done, provide the method used to generate the randomisation sequence.	lines 378-380, 391-392
		b. Describe the strategy used to minimise potential confounders such as the order of treatments and measurements, or animal/cage location. If confounders were not controlled, state this explicitly.	lines 378-381, 391-393
**Blinding**	5	Describe who was aware of the group allocation at the different stages of the experiment (during the allocation, the conduct of the experiment, the outcome assessment, and the data analysis).	N/A
**Outcome measures**	6	a. Clearly define all outcome measures assessed (e.g. cell death, molecular markers, or behavioural changes).	lines 382, 386-388, 401-403, 416-418
		b. For hypothesis-testing studies, specify the primary outcome measure, i.e. the outcome measure that was used to determine the sample size.	N/A
**Statistical methods**	7	a. Provide details of the statistical methods used for each analysis, including software used.	lines 577-582
		b. Describe any methods used to assess whether the data met the assumptions of the statistical approach, and what was done if the assumptions were not met.	N/A
**Experimental animals**	8	a. Provide species-appropriate details of the animals used, including species, strain and substrain, sex, age or developmental stage, and, if relevant, weight.	lines 378-379, 390, 404
		b. Provide further relevant information on the provenance of animals, health/immune status, genetic modification status, genotype, and any previous procedures.	lines 379-382, 390-392
**Experimental procedures**	9	For each experimental group, including controls, describe the procedures in enough detail to allow others to replicate them, including:	lines 377-388, 389-418
		a. What was done, how it was done and what was used.	lines 382-388, 393-418
		b. When and how often.	lines 378, 399, 416
		c. Where (including detail of any acclimatisation periods).	
		d. Why (provide rationale for procedures).	lines 382-388, 400-403, 416-418
**Results**	10	For each experiment conducted, including independent replications, report:	
		a. Summary/descriptive statistics for each experimental group, with a measure of variability where applicable (e.g. mean and SD, or median and range).	Fig. 1, 2, 3, 4, 5, 6, and Supplementary Fig. 5; lines 66-297
		b. If applicable, the effect size with a confidence interval.	N/A

The Recommended Set

These items complement the Essential 10 and add important context to the study. Reporting the items in both sets represents best practice.

Item		Recommendation	Section/line number, or reason for not reporting
**Abstract**	11	Provide an accurate summary of the research objectives, animal species, strain and sex, key methods, principal findings, and study conclusions.	lines 17-28
**Background**	12	a. Include sufficient scientific background to understand the rationale and context for the study, and explain the experimental approach.	lines 31-64
		b. Explain how the animal species and model used address the scientific objectives and, where appropriate, the relevance to human biology.	N/A
**Objectives**	13	Clearly describe the research question, research objectives and, where appropriate, specific hypotheses being tested.	lines 56-64
**Ethical statement**	14	Provide the name of the ethical review committee or equivalent that has approved the use of animals in this study, and any relevant licence or protocol numbers (if applicable). If ethical approval was not sought or granted, provide a justification.	lines 418-420
**Housing and husbandry**	15	Provide details of housing and husbandry conditions, including any environmental enrichment.	lines 380-382, 390-393
**Animal care and monitoring**	16	a. Describe any interventions or steps taken in the experimental protocols to reduce pain, suffering and distress.	lines 400, 416
		b. Report any expected or unexpected adverse events.	N/A
		c. Describe the humane endpoints established for the study, the signs that were monitored and the frequency of monitoring. If the study did not have humane endpoints, state this.	lines 400, 416
**Interpretation/ scientific implications**	17	a. Interpret the results, taking into account the study objectives and hypotheses, current theory and other relevant studies in the literature.	lines 300-375
		b. Comment on the study limitations including potential sources of bias, limitations of the animal model, and imprecision associated with the results.	N/A
**Generalisability/ translation**	18	Comment on whether, and how, the findings of this study are likely to generalise to other species or experimental conditions, including any relevance to human biology (where appropriate).	N/A
**Protocol registration**	19	Provide a statement indicating whether a protocol (including the research question, key design features, and analysis plan) was prepared before the study, and if and where this protocol was registered.	N/A
**Data access**	20	Provide a statement describing if and where study data are available.	lines 586-588
**Declaration of interests**	21	a. Declare any potential conflicts of interest, including financial and non-financial. If none exist, this should be stated.	line 600
		b. List all funding sources (including grant identifier) and the role of the funder(s) in the design, analysis and reporting of the study.	lines 590-594
