# Supplementary material for: Lactobacillus amylovorus extracellular vesicles mitigate mammary gland ferroptosis via the gut-mammary gland axis
Source: NPJ Biofilms Microbiomes. 2025 Jun 21;11:113. doi: 10.1038/s41522-025-00752-4 (PMC12182568; doi:10.1038/s41522-025-00752-4)

Figure 1. Oxidative Stress Induces Ferroptosis in Sow Mammary Glands and Inhibits Milk Fat Synthesis

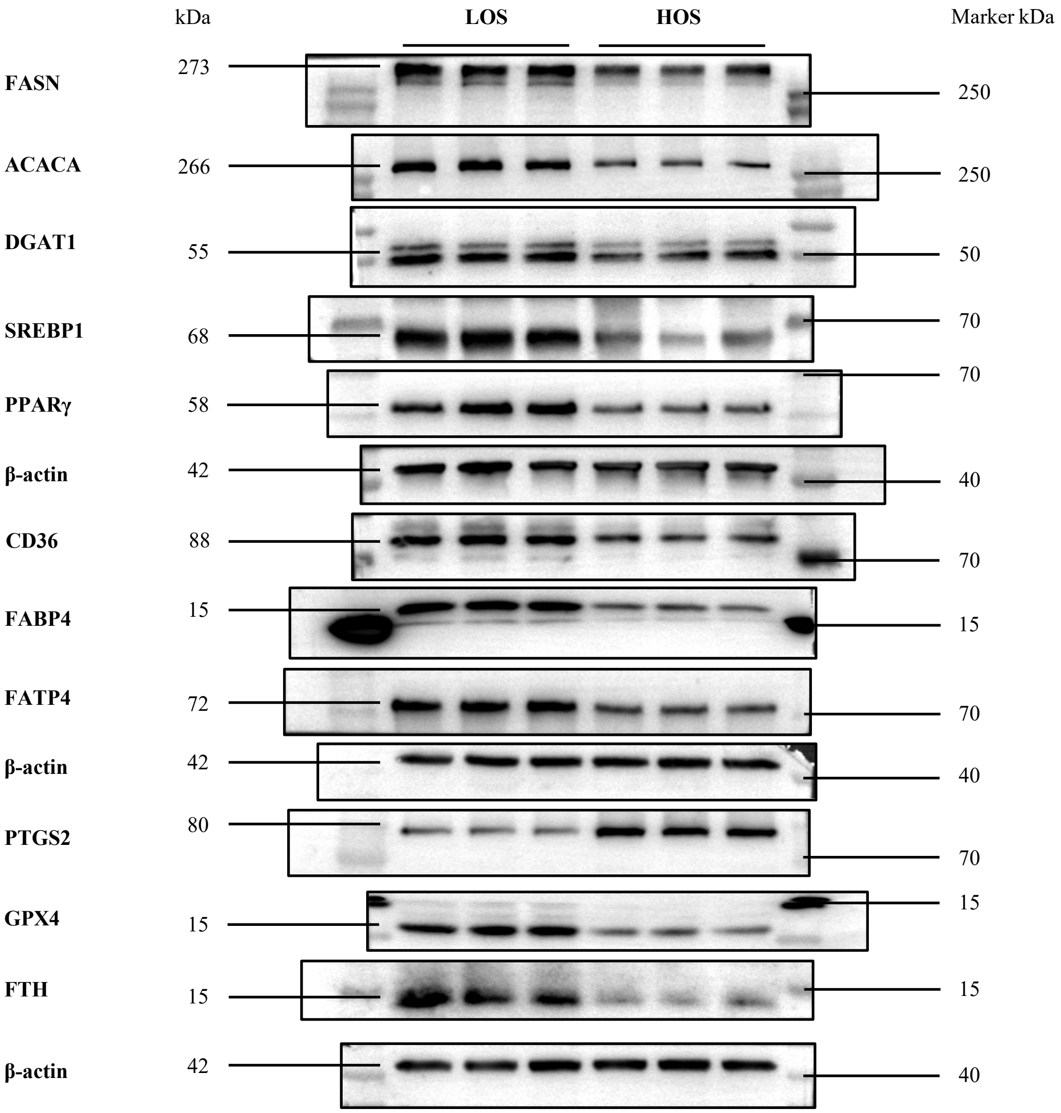

Figure 3. Impact of Maternal Gut Microbiota Transplantation on Ferroptosis in Mammary Glands and Lactation Performance

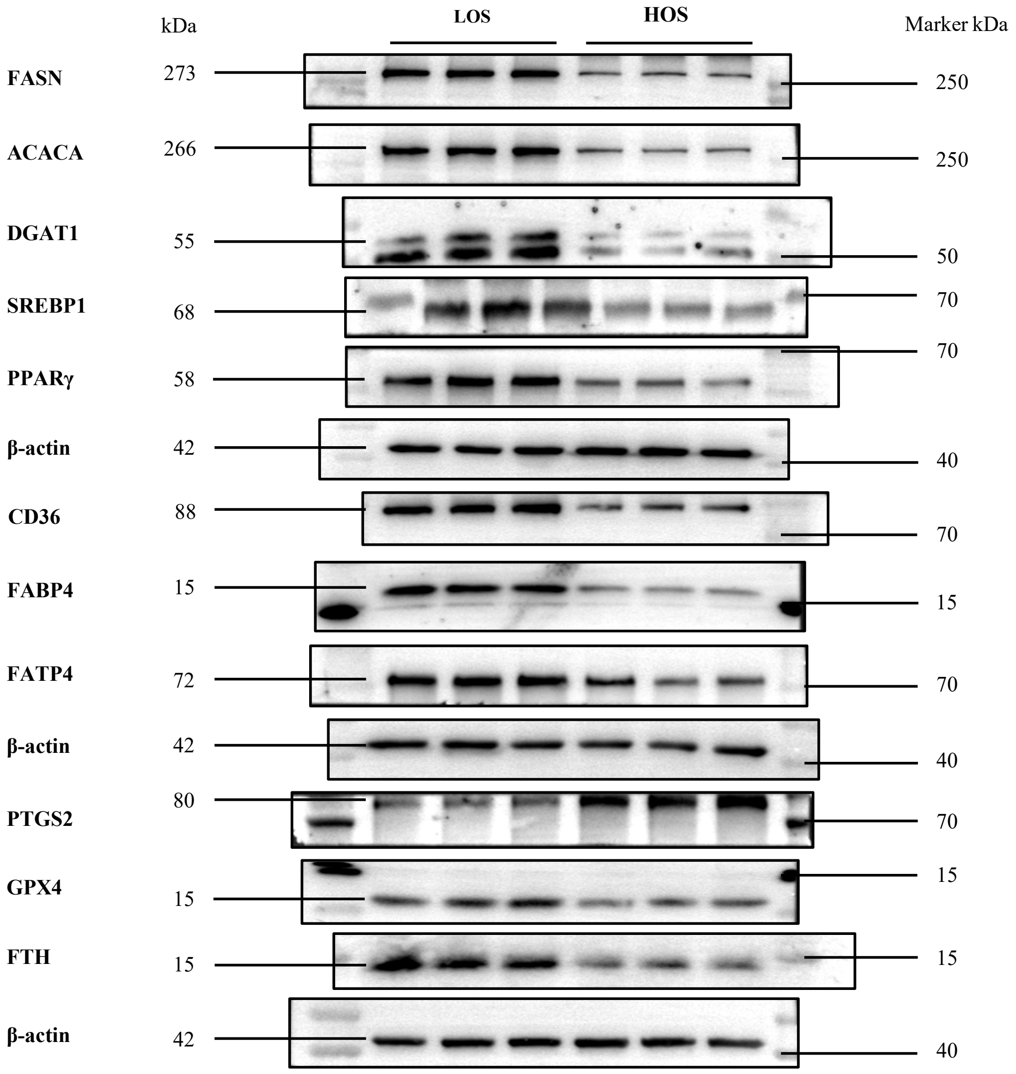

Figure 4. Maternal Microbiota Transplantation Reduces Ferroptosis in Mammary Glands and Enhances Lactation Performance under Oxidative Stress

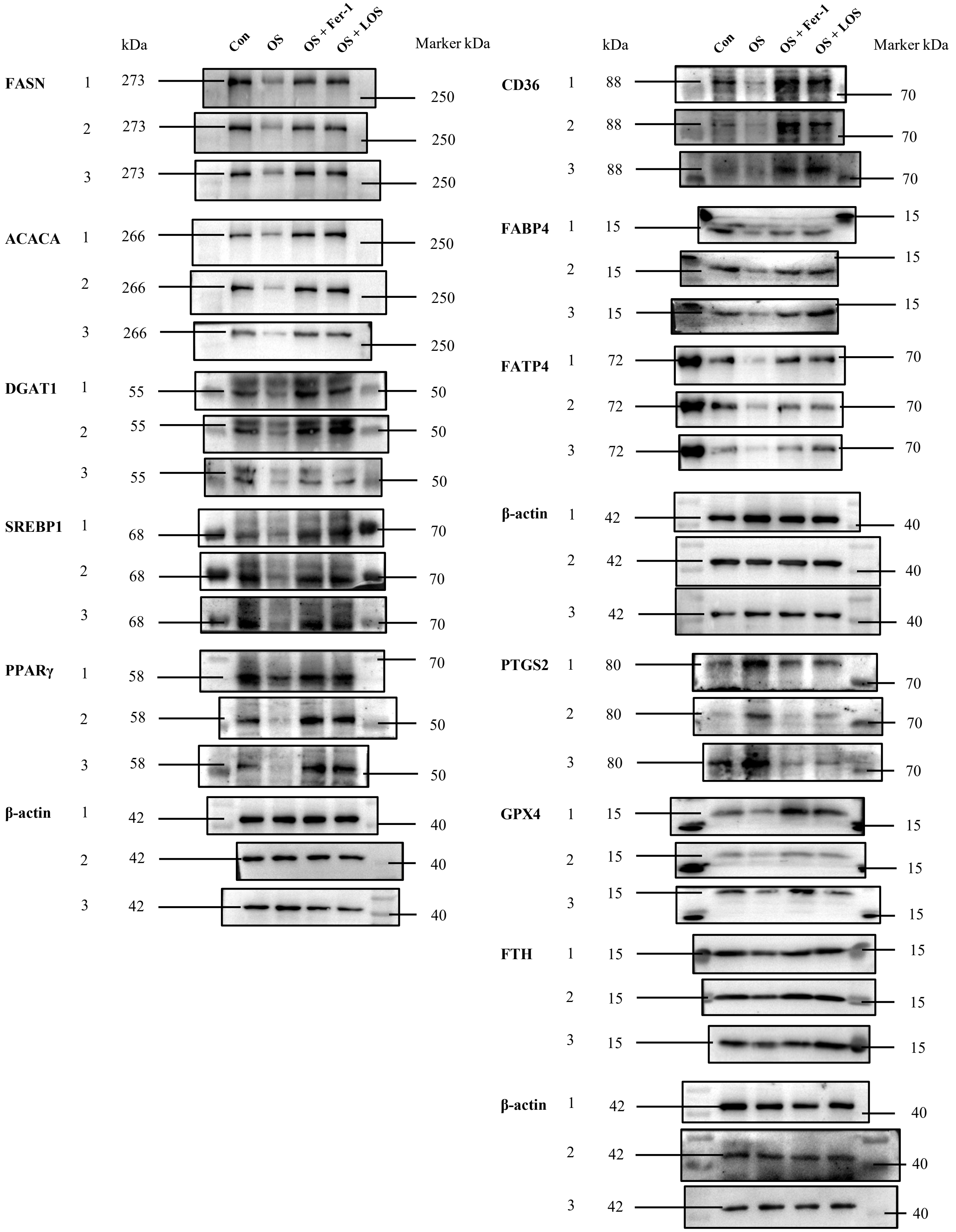

Figure 5. *Lactobacillus amylovorus* Reduces Oxidative Stress-Induced Ferroptosis in Mammary Glands and Enhances Lactation Performance via Metabolites and Extracellular Vesicles

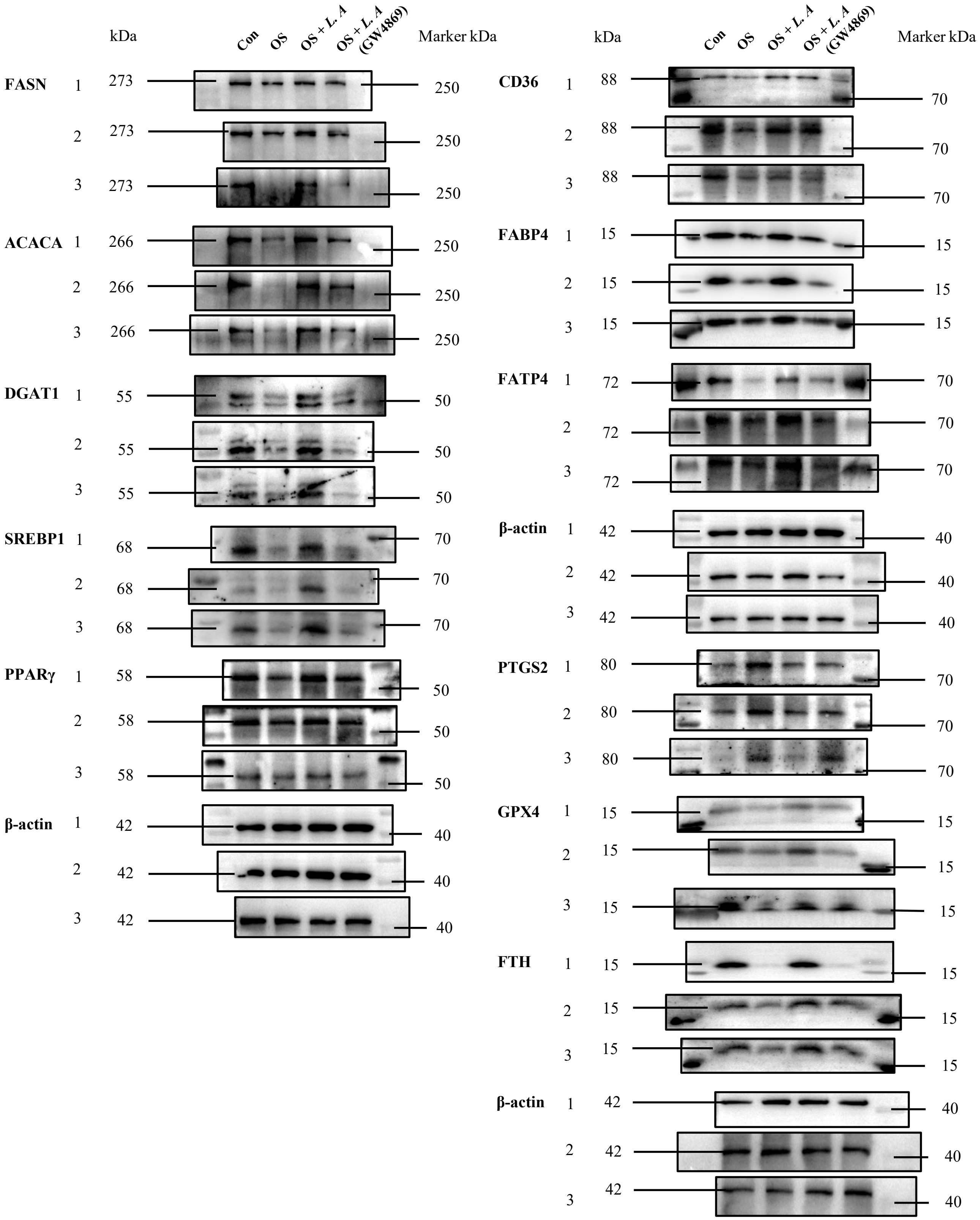

Figure 6. ***Lactobacillus amylovorus*** **Alleviates Oxidative Stress-Induced Ferroptosis in Mouse Mammary Glands and Enhances Lactation Performance via Extracellular Vesicles**

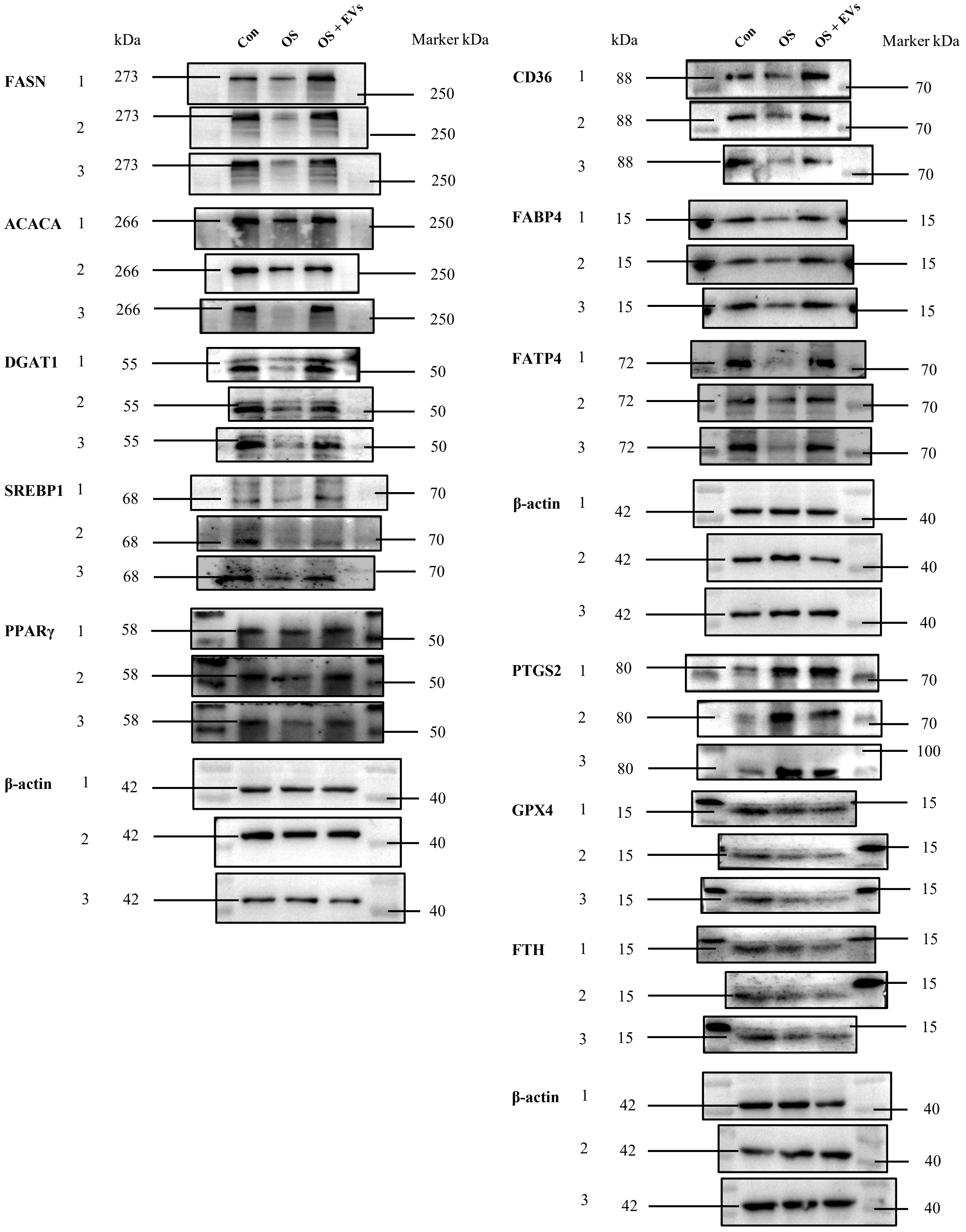

Supplement: Supplementary file 2 — Supplementary File-Western Blot Bands [file 41522_2025_752_MOESM2_ESM.pdf]
